# Synthesis, molecular docking, and in-vitro studies of pyrimidine-2-thione derivatives as antineoplastic agents via potential RAS/PI3K/Akt/JNK inhibition in breast carcinoma cells

**DOI:** 10.1038/s41598-022-26571-7

**Published:** 2022-12-22

**Authors:** Maha M. Salem, Marian N. Gerges, Ahmed A. Noser

**Affiliations:** 1 Biochemistry Division, Chemistry Department, Faculty of Science, Tanta University, Tanta, 31527 Egypt; 2 Organic Chemistry, Chemistry Department, Faculty of Science, Tanta University, Tanta, 31527 Egypt

**Keywords:** Biochemistry, Cancer, Chemical biology, Drug discovery, Molecular biology, Structural biology, Biomarkers, Medical research, Molecular medicine, Oncology, Chemistry

## Abstract

In the present investigation, derivatives from (**2**–**6**) containing pyrimidine-2-thione moiety incorporated with different heterocycles such as pyrazoline, phenyl pyrazoline, and pyrimidine were synthesized using different methods. These pyrimidine-2-thione derivatives were evaluated in-silico for their capability to inhibit the H-RAS-GTP active form protein with insight to their pharmacokinetics properties. According to our findings, compound **5a** was selected for in vitro studies as it has the in-silico top-ranked binding energy. Furthermore, compound **5a** induced apoptosis to panels of cancer cell lines with the best IC_50_ on MCF-7 breast cancer cells (2.617 ± 1.6 µM). This effect was associated with the inhibition of phosphorylated RAS, JNK proteins, and PI3K/Akt genes expression. Thus, compound **5a** has upregulated p21 gene and p53 protein levels. Moreover, **5a** arrested the cell cycle progression at the sub-G0/G1 phase. In conclusion, the synthesized compound, **5a** exhibited potent antineoplastic activity against breast cancer cell growth by targeting RAS/ PI3K/Akt/ JNK signaling cascades.

## Introduction

Breast cancer is the most frequent kind of cancer and the second-leading cause of mortality among women worldwide. Currently, the backbone of therapy for breast cancer is mainly chemotherapy, however, the majority of its chemotherapeutic medications are associated with toxicity in normal cells and drug resistance. Therefore, there is an urgent need for the development of effective, and pharmacologically safe antineoplastic agents to treat breast cancer^1^.

The pyrimidinone ring is central in novel drugs because is it known to exhibit diverse biological activities such as antiviral, antimicrobial, antioxidant, and anticancer activities^2–5^. Chalcones is also known for its wide range of biological properties, including anticancer activities. These substances are also very interesting because they can be used to create a variety of biologically active heterocycles, including azepines, pyrazolines, and flavones^6^.

Pyrazoles are an extremely important scaffold for numerous anticancer agents. The presence of the pyrazole moiety in several biologically active drugs has enabled the preparation of novel pyrazoline derivatives that can be used as anticancer, antimicrobial, antioxidant, antimalarial, and antiviral^7,8^. Here, we will focus on their anticancer activity.

The α, β-unsaturated ketones facile the synthesis of pyrimidinone derivatives through the reaction with urea or thiourea^9–12^ and pyrazoline derivatives through the reaction with hydrazine and its derivatives^13,14^.

The progression of cancer is often accompanied by the abnormality of several signaling pathways. Abnormalities in pro-survival, apoptotic, and cell regulatory pathways have become increasingly important in the pathogenesis of breast carcinogenesis^15^. Targeting these pathways with pharmacologically safe agents has been considered an appealing cancer prevention and treatment approach.

The RAS family members like H-RAS, K-RAS, and N-RAS are tiny guanosine triphosphatases (GTPases) which are the first human oncogenes that control a range of biological responses^16^. RAS proteins transmit extracellular signals to the cytoplasmic signaling network via tyrosine kinase receptors then to the nucleus which in turn promotes the transcription of genes involved in cell growth, proliferation, and death. The hyperactive RAS signaling contributes significantly to breast cancer’s growth, progression, and metastasis which is also linked to breast cancer patients' lower survival rates^17^. Thus, the effective control of oncogenic RAS is a therapeutic strategy in breast cancer^18^.

Moreover, RAS effector families have been associated with the initiation and maintenance of cancer. Therefore, it is anticipated that the inhibition of downstream proteins within these pathways could be a successful strategy for preventing RAS-mediated oncogenesis. One of the main targets of the RAS effector signaling is the RAS/PI3K/Akt pathway^19^.

PI3Ks are a family of lipid kinases that are responsible for the phosphorylation and activation of Akt^20^. Akt (protein kinase B (PKB)) is a serine-threonine protein kinase with oncogenic and antiapoptotic properties^21^. In breast cancer, the elevated expression of Akt causes normal breast cells to become neoplasm cells via the dysregulation of the cyclin-dependent kinase (CDK) inhibitor, p21^22^, and inactivation or mutant to tumor suppressor, p53 protein so that the replication of abnormal DNA is not stopped^23^.

Moreover, RAS activates the protein kinase, JNK which is known to regulate numerous physiological processes, including cell proliferation, differentiation, survival, and death. The JNK kinase family includes three proteins, namely, JNK1, JNK2 which are expressed in most tissues, and JNK3, which is expressed in the tissues of the brain, heart, and testes. JNK phosphorylates the transcription factor, c-Jun at Ser-63 and Ser-73 and these sites have been demonstrated to be crucial for the induction of c-Jun activity and cooperation with RAS in oncogenic transformation. Considering that the JNK pathway has been associated with cancer progression, RAS regulation of JNK activity may have important implications for cancer biology^24^.

In the present study, we synthesized pyrimidine-2-thione derivatives to clarify the underlying mechanisms in-silico and in-vitro by modulating the RAS/PI3K/Akt/JNK signaling pathways. This illuminates the idea of creating RAS protein inhibitors and offers a possible path as a future treatment against breast cancer.

## Materials and methods

### Chemistry

#### Chemicals and cell lines

All the chemicals purchased were of high grade from Sigma-Aldrich Chemical Co. (St. Louis, MO, USA). Doxorubicin (DOX) HCl injection was purchased from Celon Lab injectables. Human breast MCF-7 (#ATCC HTB-22), MDA-MB-231 (#ATCC HTB-26), colorectal Caco-2 (# ATCC HTB-37), and pancreatic (# ATCC CRL-1469) cancer cell lines, as well as normal human amniotic WISH (# ATCC CCL-25) cells, have been obtained by the Center of Excellence for Research in Regenerative Medicine, and its Applications, (CERMA) Alexandria University, Egypt from the American Type Culture Collection (ATCC) organization. These cells have been cultured in DMEM medium which was supplemented with 10% (v/v) fetal bovine serum (FBS) and 1% penicillin–streptomycin at 37 °C in humidified air with 5% CO_2_.

### Method A: Conventional heating

#### General method for the synthesis of compounds **1a**, **b**

Compounds **1a**, **b** were synthesized as described by Kappe^25^.

#### General method for the synthesis of chalcones (**2a, b**)

A mixture of compound **1** (25 mmol), vanillin (3.80 g, 25 mmol), and sodium hydroxide (2 g, 50 mmol) in ethanol (50 mL) were stirred at room temperature for 24 h (TLC control). Then, the reaction mixture was poured into ice water, filtered off, and dried to afford **2a, b**^26^.

##### [*E*]-1-(4-(4-(dimethylamino)phenyl)-6-methyl-2-thioxo-1,2,3,4-tetrahydropyrimidin-5-yl)-3-(4-hydroxy-3-methoxyphenyl)prop-2-en-1-one (**2a**)

Yield 92%; mp 130 °C; ^1^H NMR (400 MHz, DMSO-d_6_) δ (ppm): 9.83, 8.87 (s, 2H, 2NH), 7.73 (d, 1H, J = 7.24 Hz, CH=CH–CO), 7.47 (d, 1H, J = 7.26 Hz, CH=CH–CO), 6.53–7.20 (m, 7H, Ar–H), 5.44 (s, 1H, CH pyrimidine), 3.79 (s, 3H, OCH_3_), 3.13 (s, 1H, OH), 2.81 (s, 6H, (N–(CH3)_2_), 2.17 (s, 3H, CH_3_); ^13^C NMR (101 MHz, DMSO-d_6_) δ (ppm): 191.90, 171.23, 165.52, 151.86, 149.36, 145.20, 143.28, 130.27, 126.76, 125.55, 122.04, 119.83, 115.04, 114.40, 112.82, 110.89, 54.13, 51.55, 42.98, 15.67; IR (KBr) ν/cm^−1^: 3596 (OH), 3433 (NH), 2911 (C–H aliph), 1666 (CO), 1588 (C=C Arom); Anal. Calcd for C_23_H_25_N_3_O_3_S (423.53): C, 65.23%; H, 5.95%; N, 9.92%; S, 7.57%. Found: C, 64.83%; H, 5.73%; N, 9.68%; S, 7.25%.

##### [*E*]-3-(4-hydroxy-3-methoxyphenyl)-1-(6-methyl-4-(thiophen-2-yl)-2-thioxo-1,2,3,4-tetrahydropyrimidin-5-yl)prop-2-en-1-one (**2b**)

Yield 89%; mp 258 °C; ^1^H NMR (400 MHz, DMSO-d_6_) δ (ppm): 9.38, 8.42 (s, 2H, 2NH), 7.51 (d, 1H, J = 7.29 Hz, CH=CH–CO), 7.33 (d, 1H, J = 7.27 Hz, CH=CH–CO), 6.45–7.12 (m, 6H, Ar–H), 5.28 (s, 1H, CH pyrimidine), 3.63 (s, 3H, OCH_3_), 2.62 (s, 1H, OH), 2.19 (s, 3H, CH_3_); ^13^C NMR (101 MHz, DMSO-d_6_) δ (ppm): 192.82, 171.87, 165.23, 149.36, 148.36, 143.92, 138.56, 128.41, 126.48, 125.55, 122.97, 122.33, 120.11, 115.04, 114.76, 112.18, 55.06, 51.26, 15.09; IR (KBr) ν/cm^−1^: 3591 (OH), 3413 (NH), 2926 (C–H aliph), 1646 (CO), 1538 (C=C Arom); Anal. Calcd for C_19_H_18_N_2_O_3_S_2_ (386.49): C, 59.05%; H, 4.69%; N, 7.25%; S, 16.59%. Found: C, 58.35%; H, 4.47%; N, 7.03%; S, 16.13%.

#### General method for the cyclization of chalcones with urea (**3a, b**)

A mixture of compound **2** (10 mmol), urea (0.6 g, 10 mmol) in ethanol (20 mL), and concentrated hydrochloric acid (3 mL) were refluxed for 7 h (TLC control). The reaction mixture was then cooled. The precipitated solid was filtered off and dried to yield **3a, b**^9,10,12^.

##### 4-(4-(4-(dimethylamino)phenyl)-6-methyl-2-thioxo-1,2,3,4-tetrahydropyrimidin-5-yl)-6-(4-hydroxy-3-methoxyphenyl)pyrimidin-2(1H)-one (**3a**)

Yield 72%; mp 160 °C; ^1^H NMR (400 MHz, DMSO-d_6_) δ (ppm): 9.64, 8.96, 8.30 (s, 3H, 3NH), 6.47–7.75 (m, 7H, Ar–H), 5.44 (s, 1H, CH pyrimidine), 5.01 (s, 1H, CH pyrimidinone), 3.02 (s, 3H, OCH_3_), 2.95 (s, 6H, (N–(CH_3_)_2_)); 2.85 (s, 1H, OH); 2.23 (s, 3H, CH_3_); ^13^C NMR (101 MHz, DMSO-d_6_) δ (ppm): 175.38, 166.44, 164.58, 157.86, 154.72, 151.85, 149.00, 144.56, 133.12, 127.76, 122.05, 117.89, 115.97, 113.47, 99.80, 96.66, 57.92, 53.77, 42.33, 17.60; IR (KBr) ν/cm^−1^: 3640 (OH), 3413 (NH), 2918 (C-H Aliph), 1605 (CO), 1568 (C=N), 1521 (C=C Arom), 1270 (C=S); Anal. Calcd for C_24_H_25_N_5_O_3_S (463.55): C, 62.18%; H, 5.44%; N, 15.11%; S, 6.92%. Found: C, 61.88%; H, 5.24%; N, 14.93%; S, 6.62%.

##### 6-(4-hydroxy-3-methoxyphenyl)-4-(6-methyl-4-(thiophen-2-yl)-2-thioxo-1,2,3,4-tetrahydropyrimidin-5-yl)pyrimidin-2(1H)-one (**3b**)

Yield 69%; mp 210 °C; ^1^H NMR (400 MHz, DMSO-d_6_) δ (ppm): 10.44, 9.94, 8.93 (s, 3H, 3NH), 6.48–7.41 (m, 6H, Ar–H), 5.47(s, 1H, CH pyrimidine), 5.25 (s, 1H, CH pyrimidinone), 3.58 (s, 3H, OCH_3_), 2.67 (s, 1H, OH); 1.90 (s, 3H, CH_3_); ^13^C NMR (101 MHz, DMSO-d_6_) δ (ppm): 175.66, 166.44, 164.87, 157.86, 154.08, 150.93, 144.85, 139.77, 129.62, 128.70, 127.77, 124.25, 120.76, 118.54, 112.54, 99.81, 96.02, 59.49, 53.49, 16.02; IR (KBr) ν/cm^−1^: 3641 (OH), 3398 (NH), 2834 (C–H Aliph), 1746 (CO), 1590 (C=N), 1530 (C=C Arom), 1265 (C=S); Anal. Calcd for C_20_H_18_N_4_O_3_S_2_ (426.51): C, 56.32%; H, 4.25%; N, 13.14%; S, 15.04%. Found: C, 56.12%; H, 4.08%; N, 12.88%; S, 14.82%.

#### General method for the cyclization of chalcones with thiourea (**4a, b**)

A mixture of compound **2 (**10 mmol), thiourea (0.76 g, 10 mmol), and sodium hydroxide (0.5 g) in ethanol (25 mL) were refluxed for 8 h (TLC control). The reaction mixture was cooled, and the formed solid was filtered off and dried to yield **4a, b**^12^.

##### 4-(4-(4-(dimethylamino)phenyl)-6-methyl-2-thioxo-1,2,3,4-tetrahydropyrimidin-5-yl)-6-(4-hydroxy-3-methoxyphenyl)pyrimidine-2(1H)-thione (**4a**)

Yield 74%; mp 180 °C; ^1^H NMR (400 MHz, DMSO-d_6_) δ (ppm): 9.73, 8.95, 8.23 (s, 3H, 3NH), 6.41–7.17 (m, 7H, Ar–H), 5.39 (s, 1H, CH pyrimidine), 5.04 (s, 1H, CH=C pyrimidine), 3.58 (s, 3H, OCH_3_), 2.78 (s, 6H, (N-(CH_3_)_2_)); 2.14 (s, 1H, OH); 1.33 (s, 3H, CH_3_); ^13^C NMR (101 MHz, DMSO-d_6_) δ (ppm): 181.09, 176.95, 174.73, 163.93, 154.07, 151.56, 150.56, 143.91, 133.48, 126.47, 120.47, 116.96, 113.10, 111.52, 99.52, 92.15, 56.98, 56.33, 43.61, 16.29; IR (KBr) ν/cm^−1^: 3641 (OH), 3412 (NH), 2921 (C-H Aliph), 1604 (C=N), 1520 (C=C Arom), 1356 (C=S); Anal. Calcd for C_24_H_25_N_5_O_2_S_2_ (479.62): C, 60.10%; H, 5.25%; N, 14.60%; S, 13.37%. Found: C, 59.90%; H, 5.05%; N, 14.42%; S, 12.93%.

##### 6-(4-hydroxy-3-methoxyphenyl)-4-(6-methyl-4-(thiophen-2-yl)-2-thioxo-1,2,3,4-tetrahydropyrimidin-5-yl)pyrimidine-2(1H)-thione (**4b**)

Yield 72%; mp 220 °C; ^1^H NMR (400 MHz, DMSO-d_6_) δ (ppm): 9.38, 8.32, 7.92 (s, 3H, 3NH), 6.37–7.14 (m, 6H, Ar–H), 5.52 (s, 1H, CH pyrimidine), 5.26 (s, 1H, CH=C pyrimidine), 3.76 (s, 3H, OCH_3_), 2.33 (s, 1H, OH); 1.80 (s, 3H, CH_3_); ^13^C NMR (101 MHz, DMSO-d_6_) δ (ppm): 180.74, 176.02, 175.02, 165.23, 153.79, 150.57, 143.28, 137.56, 127.76, 127.41, 126.84, 122.69, 120.11, 116.33, 112.18, 98.53, 91.59, 56.63, 54.69, 16.31; IR (KBr) ν/cm^−1^: 3641 (OH), 3179 (NH), 2957 (C-H Aliph), 1611 (C=N), 1565 (C=C Arom), 1277 (C=S); Anal. Calcd for C_20_H_18_N_4_O_2_S_3_ (442.58): C, 54.28%; H, 4.10%; N, 12.66%; S, 21.74%. Found: C, 54.12%; H, 3.94%; N, 12.44%; S, 21.44%.

#### General method for the cyclization of chalcones with phenylhydrazine (**5a, b**)

A mixture of compound **2** (10 mmol) and phenylhydrazine (1.62 g, 15 mmol) in acetic acid (20 mL) was refluxed for 9 h (TLC control). Then, the reaction mixture was poured into ice water, filtered off, and dried to give **5a, b**^13^.

##### 4-(4-(dimethylamino)phenyl)-5-(5-(4-hydroxy-3-methoxyphenyl)-1-phenyl-4,5-dihydro-1H-pyrazol-3-yl)-6-methyl-3,4-dihydropyrimidine-2(1H)-thione (**5a**)

Yield 70%; mp 75 °C ^1^H NMR (400 MHz, DMSO-d_6_) δ (ppm): 9.76, 9.67 (s, 2H, 2NH), 6.59–7.72 (m, 12H, Ar–H), 4.97 (s, 1H, CH pyrimidine), 3.80 (t, 1H, J = 4.67 Hz, CH pyrazoline), 3.60 (s, 3H, OCH_3_), 2.98 (s, 1H, OH); 2. 70 (s, 6H, (N-(CH_3_)_2_)); 1.90 (d, 2H, J = 4.77 Hz, CH_2_); 1.20 (s, 3H, CH_3_); ^13^C NMR (101 MHz, DMSO-d_6_) δ (ppm): 175.38, 153.14, 150.92, 147.72, 146.78, 146.14, 144.85, 132.20, 129.62, 128.98, 128.05, 119.18, 118.54, 115.68, 113.47, 112.54, 110.60, 99.17, 64.28, 58.56, 54.42, 35.68, 32.18, 15.09; IR (KBr) ν/cm^−1^: 3641 (OH), 3235 (NH), 2920 (C-H Aliph), 1598 (C=N), 1516 (C=C Arom), 1258 (C=S); Anal. Calcd for C_29_H_31_N_5_O_2_S (513.65): C, 67.81%; H, 6.08%; N, 13.63%; S, 6.24%. Found: C, 67.41%; H, 5.90%; N, 13.33%; S, 5.84%.

##### 5-(5-(4-hydroxy-3-methoxyphenyl)-1-phenyl-4,5-dihydro-1H-pyrazol-3-yl)-6-methyl-4-(thiophen-2-yl)-3,4-dihydropyrimidine-2(1H)-thione (**5b**)

Yield 72%; mp 170 °C; ^1^H NMR (400 MHz, DMSO-d_6_) δ (ppm): 9.59, 8.92 (s, 2H, 2NH), 6.65–7.61 (m, 11H, Ar–H), 4.97 (s, 1H, CH pyrimidine), 3.80 (t, 1H, J = 4.62 Hz, CH- pyrazoline), 2.69 (s, 3H, OCH_3_), 2.09 (s, 1H, OH); 1.86 (d, 2H, J = 4.69 Hz, CH_2_); 1.24 (s, 3H, CH_3_); ^13^C NMR (101 MHz, DMSO-d_6_) δ (ppm): 175.73, 155.65, 150.93, 149.64, 144.65, 143.64, 139.21, 137.91, 128.98, 126.12, 125.84, 123.33, 121.04, 118.19, 114.75, 111.89, 104.60, 56.63, 54.70, 53.77, 36.61, 12.52; IR (KBr) ν/cm^−1^: 3641 (OH), 3244 (NH), 2948 (C-H Aliph), 1610 (C=N), 1500 (C=C Arom), 1260 (C=S); Anal. Calcd for C_25_H_24_N_4_O_2_S_2_ (476.61): C, 63.00%; H, 5.08%; N, 11.76%; S, 13.46%. Found: C, 62.44%; H, 4.94%; N, 11.46%; S, 13.04%.

#### General method for the cyclization of chalcones with hydrazine hydrate (**6a, b**)

A mixture of compound **2** (10 mmol) and hydrazine hydrate (1.00 g, 20 mmol) in absolute ethanol (10 mL) were refluxed for 8 h (TLC control). The formed precipitate was filtered and dried to afford **6a, b**^14^.

##### 4-(4-(dimethylamino)phenyl)-5-(5-(4-hydroxy-3-methoxyphenyl)-4,5-dihydro-1H-pyrazol-3-yl)-6-methyl-3,4-dihydropyrimidine-2(1H)-thione (**6a**)

Yield 74%; mp 190 °C; ^1^H NMR (400 MHz, DMSO-d_6_) δ (ppm): 10.28, 10.04, 9.56 (s, 3H, 3NH), 6.64–7.45 (m, 7H, Ar–H), 6.02 (s, 1H, CH-pyrimidine), 5.64 (t, 1H, J = 4.82 Hz, CH-pyrazoline), 3.54 (s, 3H, OCH_3_), 2.69 (s, 6H, (N-(CH_3_)_2_)), 2.19 (d, 2H, J = 4.84 Hz CH_2_), 2.08 (s, 1H, OH), 1.87 (s, 3H, CH_3_); ^13^C NMR (101 MHz, DMSO-d_6_) δ (ppm): 174.09, 153.14, 149.93, 148.71, 146.14, 141.99, 135.99, 131.27, 128.41, 119.47, 116.61, 114.11, 112.18, 104.60, 56.62, 53.77, 50.91, 43.33, 42.04, 16.31; IR (KBr) ν/cm^−1^: 3640 (OH), 3412 (NH), 2923 (C-H Aliph), 1610 (C=N), 1569 (C=C Arom), 1271 (C=S); Anal. Calcd for C_23_H_27_N_5_O_2_S (437.56): C, 63.13%; H, 6.22%; N, 16.01%; S, 7.33%. Found: C, 62.83%; H, 6.02%; N, 15.81%; S, 6.93%.

##### 5-(5-(4-hydroxy-3-methoxyphenyl)-4,5-dihydro-1H-pyrazol-3-yl)-6-methyl-4-(thiophen-2-yl)-3,4-dihydropyrimidine-2(1H)-thione (**6b**)

Yield 71%; mp 250 °C; ^1^H NMR (400 MHz, DMSO-d_6_) δ (ppm): 10.08, 9.73, 9.63 (s, 3H, 3NH), 6.64–7.66 (m, 6H, Ar–H), 5.89 (s, 1H, CH-pyrimidine), 5.59 (t, 1H, J = 4.62 Hz, CH-pyrazoline), 3.46 (s, 3H, OCH_3_), 2.90 (s, 1H, OH), 2.10 (d, 2H, J = 4.64 Hz CH_2_), 1.85 (s, 3H, CH_3_); ^13^C NMR (101 MHz, DMSO-d_6_) δ (ppm): 175.02, 155.65, 150.92, 145.85, 141.71, 139.49, 139.21, 125.19, 124.90, 122.05, 120.11, 115.68, 112.18, 103.60, 55.05, 53.48, 50.63, 42.04, 16.31; IR (KBr) ν/cm^−1^: 3641 (OH), 3404 (NH), 2924 (C-H Aliph), 1570 (C=N), 1521 (C=C Arom), 1276 (C=S);Anal. Calcd for C_19_H_20_N_4_O_2_S_2_ (400.52): C, 56.98%; H, 5.03%; N, 13.99%; S, 16.01%. Found: C, 56.48%; H, 4.87%; N, 13.73%; S, 15.66%.

### Method B: Ultrasonic technique

In a 100 mL round flask, a mixture of reactants was sonicated using an ultrasonic apparatus at a frequency of 60 Hz and power of 290 W for 35–45 min within a range of 75–80 °C (TLC control). The solid precipitate of the desired product was filtered off and dried under vacuum conditions.

### Simulation studies using molecular docking

The binding of the newly synthesized pyrimidine-2-thione derivatives to the target protein H-RAS GTP-active form (PDB # 5P21) was examined using molecular docking stimulators. The target protein crystal structure that has been deposited and available through worldwide protein data bank link (https://www.rcsb.org/structure/5P21) was downloaded, then rectified and optimized by eliminating co-crystallized ligand and undesired water molecules, followed by energy minimization. Additionally, Chemdraw Ultra 8.0 (https://en.freedownloadmanager.org/users-choice/Chemdraw_Ultra_8.0.html) was used to create the two-dimensional (2D) structures of the prepared analogs. These structures were later translated to motif files as three-dimensional (3D). The interaction between the target protein and ligands was examined using Molegro Virtual Docker (http://molexus.io/molegro-virtual-docker/). The Discovery Studio 3.5 tool application (https://discover.3ds.com/discovery-studio-visualizer-download) was used to display the intermolecular interactions between the newly synthesized pyrimidine-2-thione derivatives and the target protein^27–30^.

#### ADMET in-silico prediction

The Swiss Institute of Bioinformatics' online tool SwissADME (http://www.swissadme.ch/) was used to examine the pharmacokinetics and drug-likeness prediction of the newly synthesized pyrimidine-2-thione derivatives. SwissADME's SMILES generator was used to transform the compound's 2D structural model into SMILES, which was then analyzed to determine the compound's different ADMET qualities in terms of pharmacokinetics and drug-likeness^31^.

### Biology section

#### Measurement of cell viability by MTT

3-(4,5-dimethylthiazol-2-yl)-2,5-diphenyltetrazolium bromide (MTT) assay was used to measure cell viability according to a prior study^32^. The cells were grown in 96-well plates at a density of 1 × 10^4^ cells/well, treated for 48 h with varied **5a** (0–200 µM) or the reference drug DOX (0–100 µM) dosages, and then exposed to analysis. The culture medium was taken out after 48 h of incubation, and the cells were given two gentle washes with 1× ice-cold phosphate-buffered saline (PBS). The MTT (10×) has been diluted to a 1× solution in the culture medium (1 volume 10× MTT in 9 volumes medium) and 200 μl was added to each well. The microplate has been kept in a cell culture incubator at 37 °C for 4 h. Afterthat, 180 μl of the MTT solution was taken out and 100 μl of DMSO was put to each well. The microplate was incubated for 15 min with shaking at room temperature. The absorbance of each well was ultimately measured at 630 nm using a Model 680 microplate reader (Bio-Rad, CA, USA)^33^.

#### Cell morphology

In a six-well plate, 1 × 10^5^ MCF-7 cells were seeded and incubated for 24 h then treated with the IC_50_ dose of the selected compound. Using an inverted light microscope (Olympus, USA), the morphological alterations of the treated and untreated cells were evaluated and documented after 48 h of incubation^34^.

#### Cell cycle analysis by flow cytometry

Using flow cytometry, cell cycle analysis was carried out via a documented method of^35^ with a few changes. After trypsinization, the MCF-7 cells were centrifuged at 4500 rpm for 5 min, followed by two washings, resuspension in 1× PBS, fixation with ice-cold absolute ethanol, and incubation at 20 °C for 24 h. Following two PBS washes, the cells were then resuspended in (0.05 mg/mL) propidium iodide (PI)^32^ solution containing 50 µL (0.2 mg/mL) of RNase A, and 0.1% v/v Triton X-100 in PBS. Then, the cells were incubated at room temperature for 30–60 min in the dark. Finally, the pellet was washed with 1⤫ PBS and resuspended in 300 µL 1× PBS, and subjected to analysis on Accuri C6 flow cytometer (Becton Dickinson, Franklin Lake, BD, USA) equipped with PE X FL2 channels^33^.

#### RT-PCR analysis

The total RNA was isolated from the MCF-7 cells using Trizol (Cat# R2072, ZYMO RESEARCH CORP. USA) according to the manufacturer’s instructions. Its quantity and quality were assessed using a Beckman dual spectrophotometer^28^. The cDNA was performed using a miScript Reverse Transcription Kit (QIAGEN) Kit (218061) and a miScript SYBR Green PCR Kit was used for mRNA detection with quantification (218073). Reverse transcription was running on a StepOne system (Applied Biosystems, Foster City, CA, USA) in a total volume of 10 μL containing 5 μL of the miScript RT enzyme and 10 μL of the total RNA. Real-time PCR for mRNA was performed on the same system in a total volume of 20 μL containing 10 μL of a 2× QuantiTect^®^ SYBR Green PCR Master Mix and 10 ng of the cDNA template. The PCR thermal cycling conditions for the mRNA were 2 min at 50 °C, 30 s at 95 °C, 40 cycles of 95 °C for 5 s, and 60 °C for 34 s. The expression of the mRNA target genes was normalized to the expression of human GAPDH quantified using the 2^−ΔΔCt^ method^36^. Table 1 contains a list of the primers used in the research.

**Table 1:** Primer sequences used in RT-PCR.

Genes	Forward sequence	Reverse sequence	Gene accession number
*PI3K*	5′TGCTATGCCTGCTCTGTAGTGGT3′	5′GTGTGACATTGAGGGAGTCGTTG3′	NM_181524.2
*Akt*	5′GTGCTGGAGGACAATGACTACGG3′	5′AGCAGCCCTGAAAGCAAGGA3′	XM_047431075.1
*p21*	5′TGTCCGTCAGAACCCATGC3′	5′AAAGTCGAAGTTCCATCGCTC3′	NM_001374511.1
*GAPDH*	5′GAGAGACCCTCACTGCTG3′	5′GATGGTACATGACAAGGTGC3′	NM_001357943.2

#### Immunoblotting analysis

Western blot analysis was conducted according to^37^. The ReadyPrepTM protein extraction kit (total protein) provided by Bio-Rad Inc (Cat #163-2086) was used to isolate proteins according to the manufacturer’s instructions. The Bradford protein assay kit (SK3041) from Bio Basic Inc. (Markham Ontario L3R 8T4 Canada) was then used to perform quantitative protein analysis. Afterward, a 20 μg protein concentration of each sample was subjected to 12% sodium dodecyl sulfate–polyacrylamide gel electrophoresis (SDS-PAGE), followed by transfer to polyvinylidene fluoride (PVDF) membranes (BioRad). Polyacrylamide gels were performed using the TGX Stain-Free™ FastCast™ Acrylamide Kit (SDS-PAGE) provided by Bio-Rad Laboratories Inc. (Cat # 161-0181). The SDS-PAGE TGX Stain-Free FastCast was prepared according to the manufacturer's instructions. The membrane was blocked in tris-buffered saline with Tween 20 (TBST) buffer and 3% bovine serum albumin (BSA) for 1 h at room temperature followed by probing with primary antibodies against phospho-RAS-GRF1 (Ser916) (1: 1000, Cell Signaling Technology, #3321), phospho-SAPK/JNK (Thr183/Tyr185) (1:1000, Cell Signaling Technology, #9251), p53 (1:1000, Cell Signaling Technology, #9282) and β-actin (1:3000, Cell Signaling Technology, #8457). The primary antibodies were diluted in TBST according to the manufacturer’s recommendations and incubated against the blotted target protein overnight at 4 °C in each primary antibody solution. The blot was rinsed with TBST 3–5 times for 5 min. The membrane was incubated with the horseradish peroxidase (HRP)-conjugated secondary antibody (Goat anti-rabbit IgG- HRP-1 mg Goat mab-Novus Biologicals) solution for 1 h at room temperature and then washed with TBST. The chemiluminescent substrate (Clarity TM Western ECL substrate Bio-Rad cat#170-5060) was applied to the blot according to the manufacturer’s recommendation and the chemiluminescent signals were captured using a charge-coupled device (CCD) camera-based imager. Image analysis software was utilized to compare the target protein’s band intensity to those of the control sample β-actin by protein normalization on the (ChemiDoc MP imager (https://www.bio-rad.com/en-us/product/chemidoc-mp-imagingsystem?ID=NINJ8ZE8Z).

#### Statistical analysis

Data have been analyzed using the GraphPad Prism software (San Diego, CA, USA) (GraphPad Prism 6, https://www.graphpad.com/scientific-software/prism/). The experimental data are expressed as mean ± SE. The significance of the difference between the treated and the control groups were analyzed using the t-test to compare these groups. The acceptable significance was recorded when the *p-*value was < 0.05.

## Results and discussion

### Chemistry of the synthesized compounds

**Figure 1:**
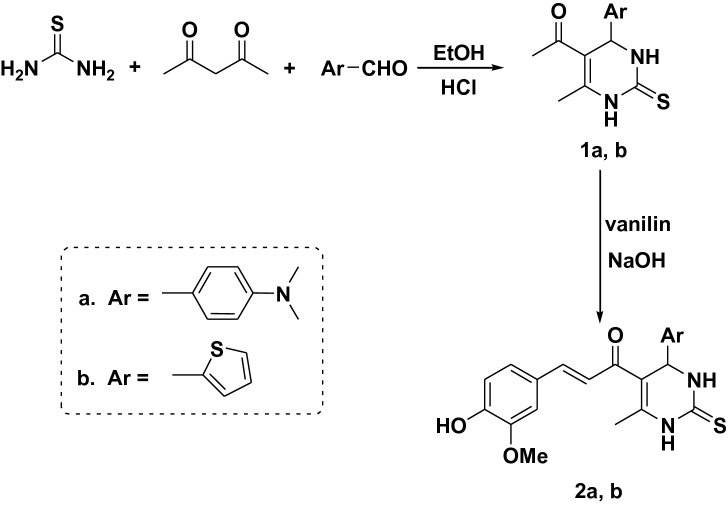
Synthesis pathway of compounds (**1**, **2**).

A one-pot three component reaction from 4-(dimethyl amino) benzaldehyde or thiophene 2-carboxyladehyde, acetylacetone and thiourea in an acidic medium led to the formation of 5-acetyl-4-(4-(dimethylamino)phenyl)-6-methyl-2-thioxo-1,2,3,4-tetrahydropyrimidine (**1a**) and 5-acetyl-6-methyl-4-(thiophen-2-yl)-2-thioxo-1,2,3,4-tetrahydropyrimidine (**1b**) respectively with high yield (Fig. 1).

Moreover, the condensation of compounds **1a, b** with vanillin in the ethanolic solution of sodium hydroxide yielded compounds **2a, b** respectively (Fig. 1), whose structures were confirmed via elemental analysis and spectral data. The IR spectrum of compounds **2a, b** exhibited absorption bands at 3596 cm^−1^ and 3591 cm^−1^ respectively characteristics for the OH group that resonated as a singlet signal at *δ* 3.13 and *δ* 2.62 respectively in the ^1^H NMR spectrum. Additionally, compounds **2a, b** showed singlet signals at *δ* 3.79 and *δ* 3.63 respectively corresponding to methoxy protons (OCH_3_). The ^13^C NMR spectrum of compounds **2a, b** displayed signals at *δ* 54.13 and 55.06 respectively owing to the methoxy group.

**Figure 2:**
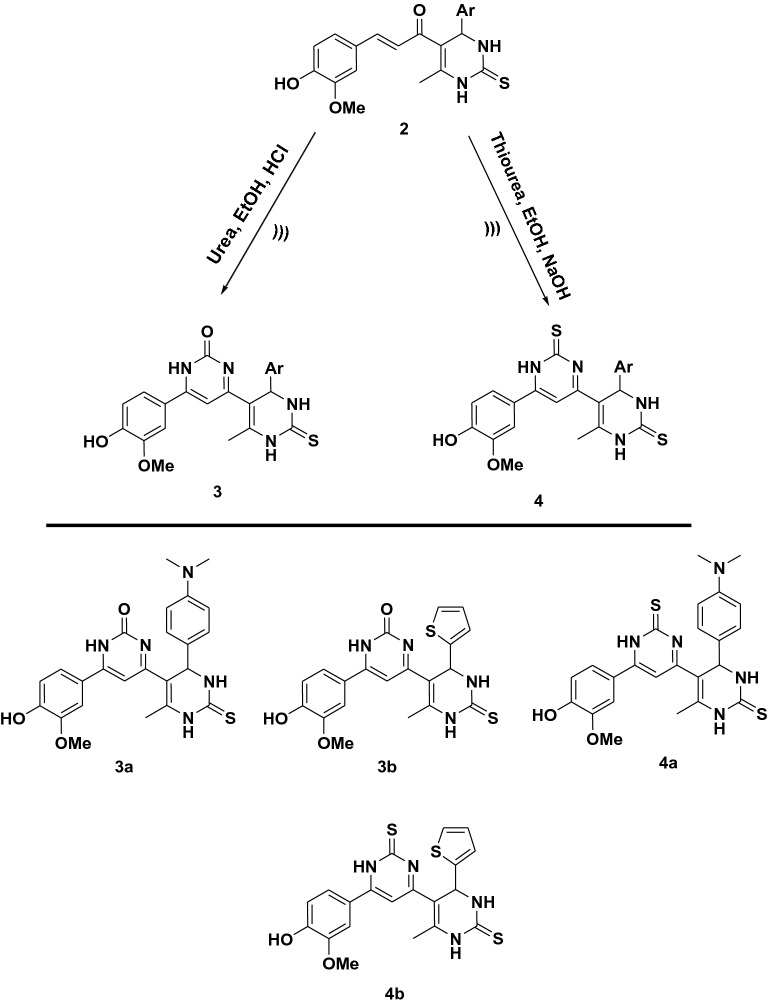
Synthesis pathway of compounds (**3**, **4**).

The condensation of chalcones **2a, b** with urea in boiling ethanolic hydrochloric acid afforded **3a, b **(Fig. 2). The IR spectrum of these compounds showed significant absorption bands at 1568–1590 cm^−1^ characteristic of C=N group, absorption band in the region within the range of* ν* 1605–1764 cm^−1^ corresponding to (C=O) group and absorption bands at 3640–3641 cm^−1^ characteristic of the OH group. The ^1^H NMR spectrum of compound **3a** showed singlet signal at *δ* 5.01 ppm ascribable to CH of new pyrimidinone ring, and a new singlet signal at *δ* 9.64 ppm ascribable to NH pyrimidinone. Its ^13^C NMR spectrum revealed signal at *δ* 149.00 attributed to C–NH pyrimidinone, *δ* 164.58 attributed to C=O pyrimidinone and *δ* 166.44 due to C=N pyrimidinone. Moreover, the ^1^H NMR spectrum of compound **3b** exhibited singlet signal at *δ* 5.25 ppm due to CH pyrimidinone and singlet signal at *δ* 10.44 ppm due to NH pyrimidinone. The ^13^C NMR spectrum exhibited two signals at *δ* 164.87 and 166.44 attributed to C=O and C=N of pyrimidinone respectively.

Similarly, chalcones **2a, b** reacted with thiourea in boiling ethanolic sodium hydroxide solution to afford **4a, b **(Fig. 2). The ^1^H NMR spectrum of compound **4a** exhibited singlet signal at* δ* 5.04 ppm due to CH of the new pyrimidine ring and singlet signals at *δ* 9.73 ppm attributed to NH proton in the new pyrimidine ring. Its ^13^C NMR spectrum revealed novel three signals at *δ* 163.93, 174.73 and 181.09 respectively attributed to C–NH, C=N and C=S of new pyrimidine ring. The ^1^H NMR spectrum of compound **4b** exhibited singlet signal at* δ* 5.26 ppm due to CH proton of the new pyrimidine ring and singlet signals at *δ* 9.38 ppm for NH proton. Its ^13^C NMR spectrum exhibited three signals attributed to C–NH, C=N and C=S groups in pyrimidine ring at *δ* 165.23, 175.02 and 180.74 respectively.

**Figure 3:**
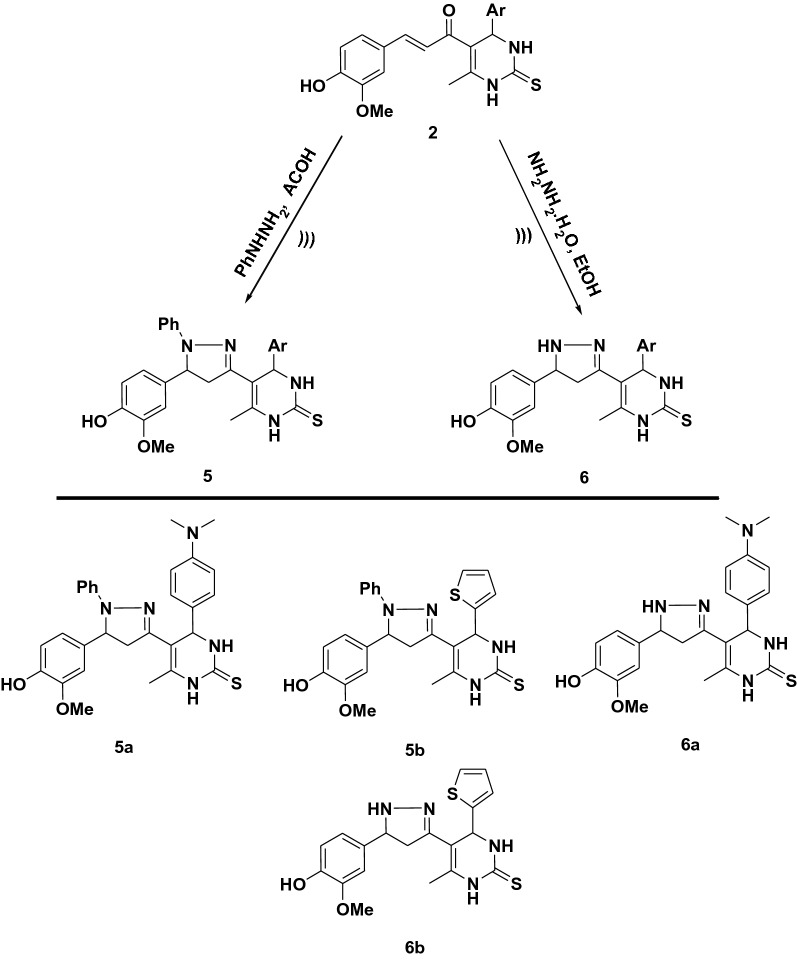
Synthesis pathway of compounds (**5**, **6**).

The cyclo-condensation of chalcones **2a, b** with phenyl hydrazine in acetic acid led to the formation of the phenyl pyrazoline derivative **5a, b **(Fig. 3). The ^1^H NMR spectra of compound **5a** offered a new doublet signal at *δ* 1.90 ppm corresponding to CH_2_ pyrazoline and a new triplet signal at *δ* 3.80 attributed to CH pyrazoline. Its ^13^C NMR spectrum exhibited three new signals at δ 32.18, 58.56 and 147.72 attributed to CH, CH_2_ and C=N of pyrazoline respectively. The ^1^H NMR spectra of compound **5b** showed doublet signal at *δ* 1.86 ppm attributed to CH_2_ pyrazoline and triplet signal at *δ* 3.80 due to CH pyrazoline. Its ^13^C NMR spectrum showed three new signals at δ 36.61, 54.70 and 149. 64 due to CH, CH_2_ and C=N respectively.

Further modification of compounds **2a, b** with hydrazine hydrate led to the formation of pyrazoline derivative **6a, b **(Fig. 3). The ^1^H NMR spectrum of compound **6a** exhibited a new doublet signal at *δ* 2.19 ppm corresponding to the CH_2_ pyrazoline, a new triplet signal at *δ* 5.64 attributed to CH pyrazoline and new singlet signals at *δ* 10.28 ppm ascribable to NH pyrazoline. Its ^13^C NMR spectrum revealed new three signals at *δ* 43.33, 50.91 and 146.14 attributed to CH_2_, CH and C=N of pyrazoline ring respectively. Moreover, the ^1^H NMR spectrum of compound **6b** showed doublet signal at *δ* 2.10 ppm for CH_2_ pyrazoline, triplet signal at *δ* 5.59 attributed to CH pyrazoline and singlet signals at *δ* 10.08 ppm ascribable to NH pyrazoline. Its ^13^C NMR spectrum showed three new signals at *δ* 42.04, 50.63 and 145.85 due to CH_2_, CH and C=N of pyrazoline ring respectively.

Ultrasonic irradiation has been determined as the most eco-friendly technique that can be used to accelerate the reaction rate and ensure superior yield during organic synthesis^38^. Therefore, a series of compounds **3**–**6** were synthesized via ultrasonic irradiation and afforded the same synthesized compounds obtained from the conventional method (melting point, mixed melting point, TLC, and spectral analyses) at higher yields in a short time (Table 2).

**Table 2:** Experimental conditions for compounds **(3–6)** by conventional and ultrasonic methods.

Compounds	Conventional method		Ultrasonic technique (75–80 °C)	
	Time (h)	Yield %	Time (min)	Yield %
3a	7	72	35	86
3b	7	69	35	82
4a	8	74	40	89
4b	8	72	40	87
5a	9	70	45	86
5b	9	72	45	88
6a	8	74	40	89
6b	8	71	40	87

## study

### Molecular docking study (in-silico)

**Figure 4:**
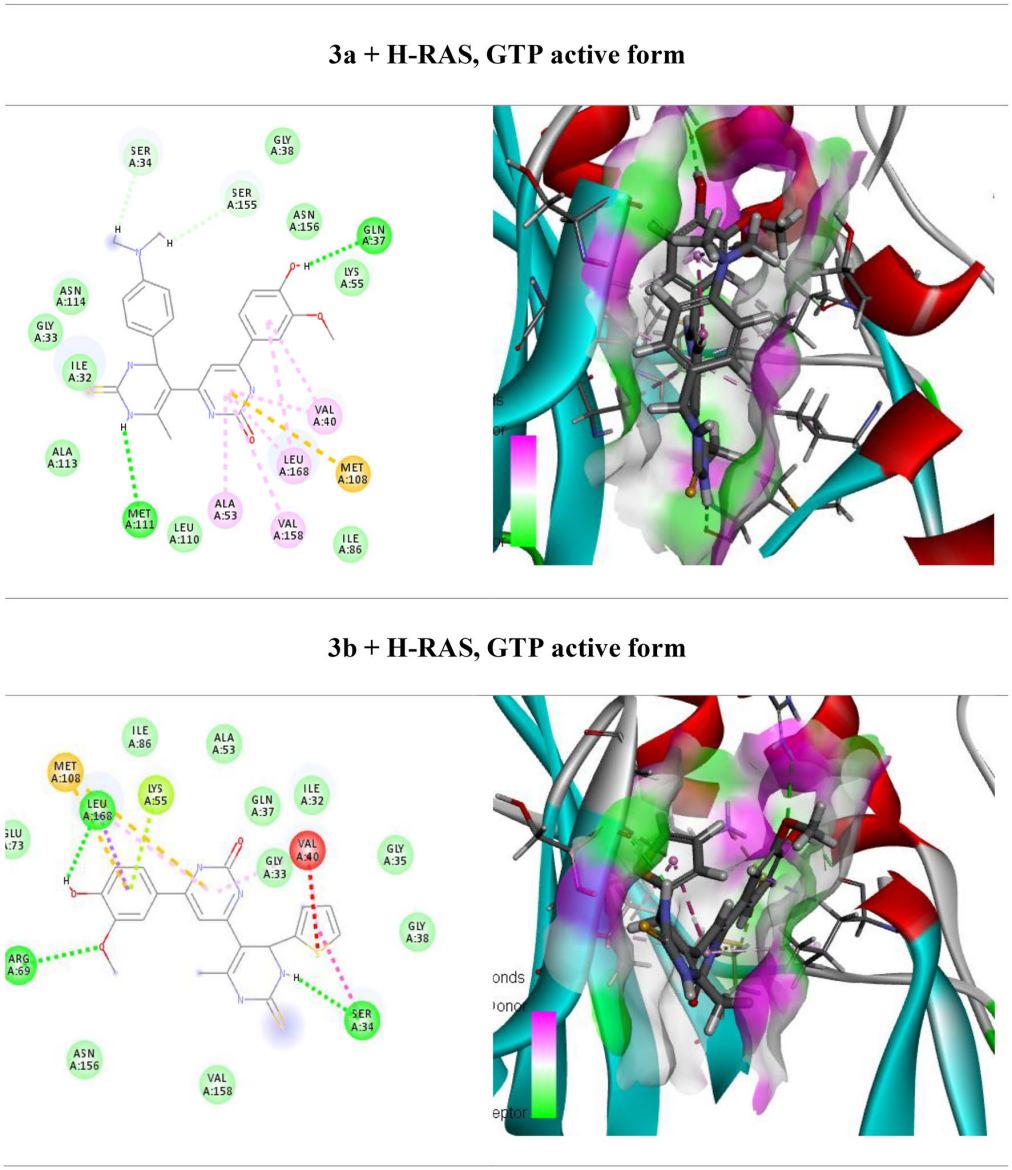

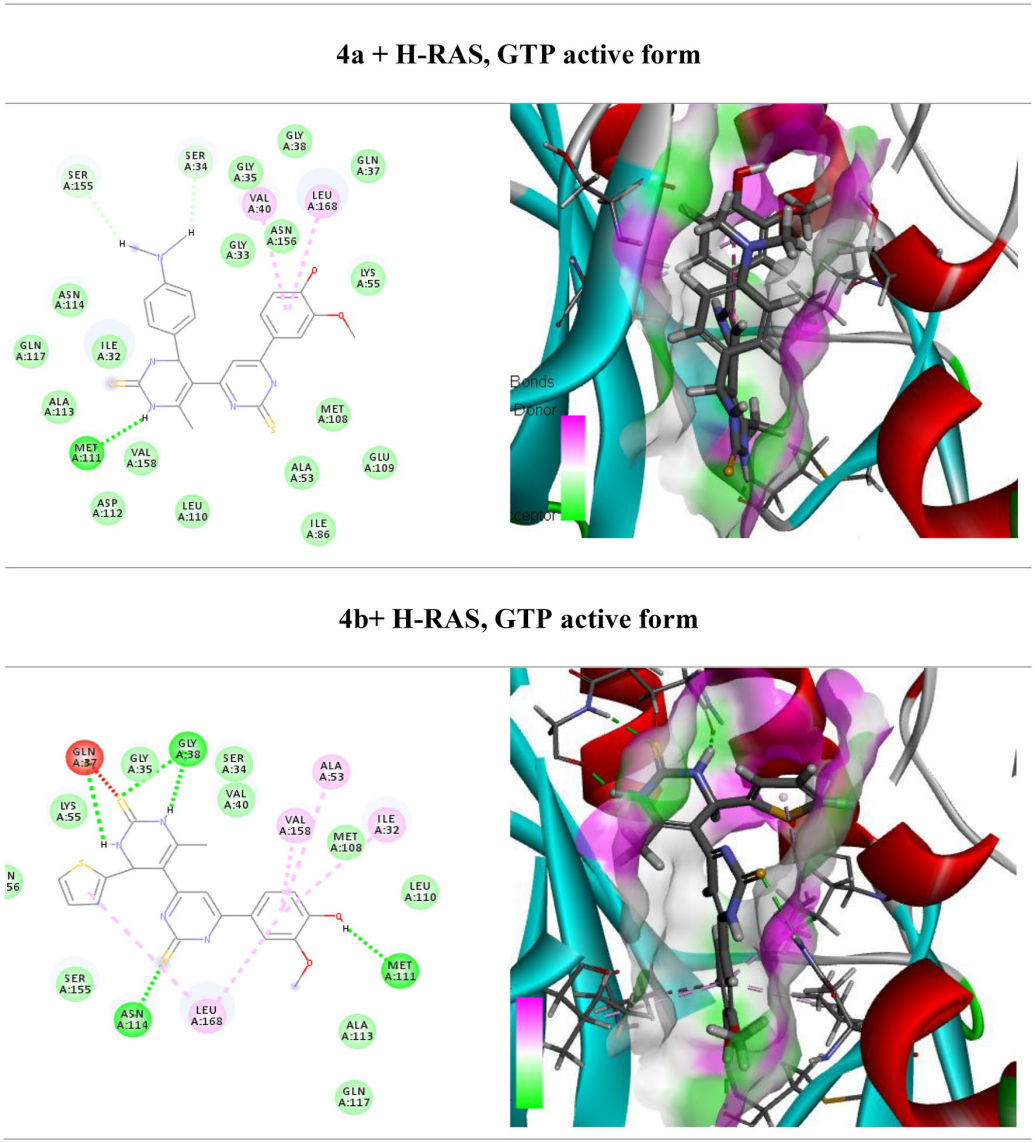

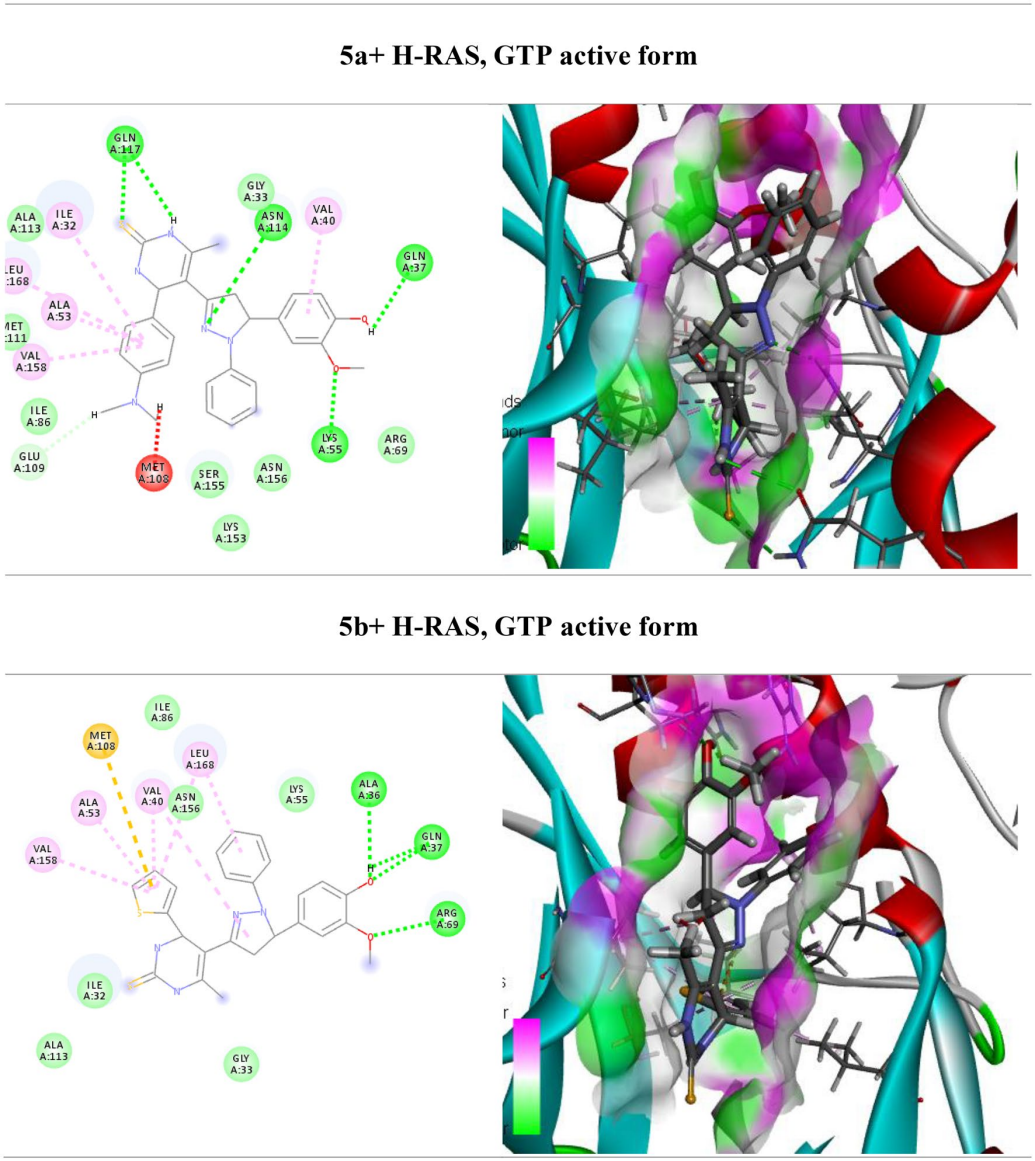

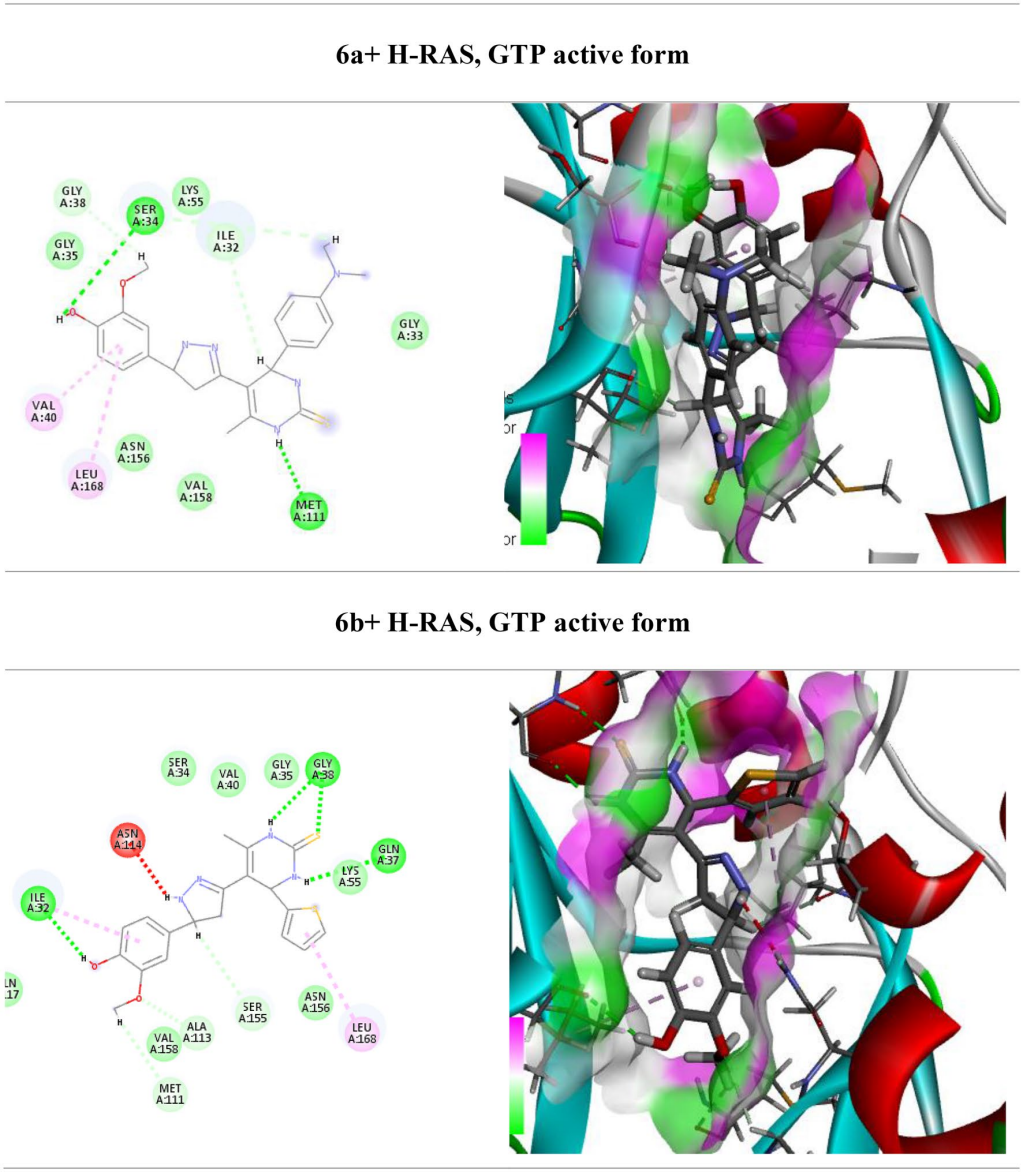
(Left side) 2D and (right side) 3D represent the interactions of the docked novel synthesized pyrimidine-2-thione derivatives with amino acid residues of the H-RAS target protein.

One of the main signaling pathways linked to tumor development is RAS/JNK and RAS/PI3K/Akt. As a result, molecular docking was used in the current study to investigate the potential H-RAS novel drug candidates. The docking scores of newly synthesized compounds against H-RAS, GTP active form target protein ranged from -5.6 to − 11.16 kcal/mol, respectively. According to the results of the molecular docking simulations, compound **5a** seemed to have the maximum binding energy with a value equal − 11.16 kcal/mol against the target H-RAS, GTP active form protein (Table 3, Fig. 4). Whereas compound **5a** docked to the protein H-RAS, GTP active form through H-bonds and π-cation interactions with the amino acid residues, Gln 117, Asn 114, Val 40, Gln 37, Lys 55, Met 108, Val 158, Ala 53, Leu 168, Ile 32 and Glu 109.

**Table 3:** Calculated docking scores (in kcal/mol) of the compounds with the target protein.

Compds	H-RAS, GTP active form		
	Docking score (ΔG_bind_)	Docked complex (amino acid–ligand) interactions	Distance [Å]
**3a**	− 7.9	Ser 34—compound **3a**	2.12
		Ser 155—compound **3a**	2.53
		Gln 37—compound **3a**	3.29
		Val 40—compound **3a**	2.92
		Met 108—compound **3a**	3.29
		Leu 168—compound **3a**	2.68
		Val 158—compound **3a**	2.65
		Ala 53—compound **3a**	3.03
		Met 111—compound **3a**	2.43
		Ile 32—compound **3a**	2.65
**3b**	− 5.6	Met 108—compound **3b**	3.00
		Leu 168—compound **3b**	3.46
		Lys 55—compound **3b**	3.29
		Arg 69—compound **3b**	2.78
		Val 40—compound **3b**	2.63
		Ser 34 —compound **3b**	2.48
		Ile 32—compound **3b**	2.25
**4a**	− 8.8	Ser 34—compound **4a**	3.02
		Ser 155—compound **4a**	2.99
		Val 40—compound **4a**	3.83
		Leu 168—compound **4a**	2.73
		Met 111—compound **4a**	2.85
		Ile 32—compound **4a**	2.70
**4b**	− 7.36	Gln 37—compound **4b**	2.65
		Gly 38—compound **4b**	3.31
		Val 158—compound **4b**	2.75
		Ala 53—compound **4b**	3.10
		Ile 32—compound **4b**	2.45
		Met 111—compound **4b**	2.68
		Leu 168—compound **4b**	3.57
		Asn 114—compound **4b**	3.49
**5a**	− 11.16	Gln 117—compound **5a**	3.17
		Asn 114—compound **5a**	3.04
		Val 40—compound **5a**	2.99
		Gln 37—compound **5a**	3.09
		Lys 55—compound **5a**	2.72
		Met 108—compound **5a**	2.53
		Val 158—compound **5a**	2.98
		Ala 53—compound **5a**	3.05
		Leu 168—compound **5a**	2.68
		Ile 32—compound **5a**	3.32
		Glu 109—compound **5a**	3.51
**5b**	− 6.86	Val 158—compound **5b**	3.29
		Ala 53—compound **5b**	3.25
		Met 108—compound **5b**	3.08
		Val 40—compound **5b**	3.12
		Leu 168—compound **5b**	3.37
		Ala 36—compound **5b**	2.59
		Val 158—compound **5b**	2.48
		Arg 69—compound **5b**	2.44
**6a**	− 9.74	Ser 34—compound **6a**	3.00
		Gly 38—compound **6a**	2.99
		Ile 32—compound **6a**	2.53
		Met 111—compound **6a**	2.42
		Leu 168—compound **6a**	3.29
		Val 40—compound **6a**	3.04
**6b**	− 7.07	Gly 38—compound **6b**	2.93
		Gln 37—compound **6b**	3.29
		Leu 168—compound **6b**	3.29
		Ser 155—compound **6b**	2.22
		Ala 113—compound **6b**	2.46
		Met 111—compound **6b**	2.58
		Ile 32—compound **6b**	3.04
		Asn 114—compound **6b**	3.32

#### Studies on ADME/T and the Lipinski rule

To certify a medicine and its efficacy as a top candidate against any disease, ADME/T is necessary. The partition coefficient (cLogP), donor hydrogen-bond, and drug similarity were all calculated using in-silico Physio-chemical methods, and they were all assessed using Lipinski's rule of five^39^. Also, studies on bioavailability and pharmacokinetics have been carried out to perform such clinical trials on these novel synthesized pyrimidine-2-thione derivatives. The topological polar surface (TPSA) must be less than < 140 Å_2_ for great oral bioavailability^40^, our results showed good oral bioavailability whereas the TPSA range = 69.12–99.27 thus, all compounds follow the Lipinski rule. Moreover, the results elucidated that all compounds showed acceptable gastrointestinal absorptions and have no BBB and Caco-2 permeability except for compounds **4a** and **4b** which showed high Caco-2 permeability.

A drug candidate must first pass a toxicity risk assessment to be considered for drug development. Low toxicity and side effects are indicative of a medicine's high therapeutic index. Due to this, an in-silico toxicity analysis was carried out, with the outcomes displayed in Table 4. Surprisingly, none of the substances proved carcinogenic.

**Table 4:** ADMET and Pharmacokinetic properties of the compounds.

Compounds/parameters	Molecular weight (g/mol)	Blood–brain barrier (log BBB)	Caco-2 permeability (Caco2 +)	%Human intestinal absorption (HIA +)	TPSA A^2^	logp	HBA	HBD	N rotatable	GI absorption	Oral rat acute toxicity (LD50)	AMES toxicity	Carcinogenicity
Acceptable ranges	≤ 500	> 0.3 great < − 1 poor	> 0.9 great	> 80% high < 30% low	≤ 140	< 5	2.0–20.0	0.0–6.0	≤ 10		Up to 3 mol/kg	Nontoxic	Noncarcinogenic
**3a**	476.627	− 0.90	0.555	88.106	69.12	2.97	4	4	5	High	2.54	Nontoxic	Noncarcinogenic
**3b**	426.523	− 1.38	0.42	82.633	99.27	2.73	6	4	4	High	2.76	Nontoxic	Noncarcinogenic
**4a**	479.62	− 1.40	1.284	89.701	85.44	3.37	3	4	5	High	2.49	Nontoxic	Noncarcinogenic
**4b**	442.58	− 1.52	1.208	87.711	82.20	3.18	3	4	4	High	2.42	Nontoxic	Noncarcinogenic
**5a**	513.667	− 0.589	0.628	90.035	72.36	2.47	7	3	6	High	2.60	Nontoxic	Noncarcinogenic
**5b**	476.627	− 0.90	0.555	88.106	69.12	3.30	3	3	5	High	2.54	Nontoxic	Noncarcinogenic
**6a**	437.569	− 0.73	0.878	87.869	81.14	3.16	3	4	5	High	2.60	Nontoxic	Noncarcinogenic
**6b**	400.52	− 0.85	0.813	85.941	77.91	2.86	3	4	4	High	2.55	Nontoxic	Noncarcinogenic

TPSA: total polar surface area, GI: Gastrointestinal, BBB: Blood brain barrier, HBA: Hydrogen bond acceptor, HBD: Hydrogen bond donor, Logp: octanol/water partition coefficient.

Additionally, an AMES toxicity analysis was conducted, and all compounds **(3a-6b)** tested negative, indicating that they have no mutagenic effects. Computed LD_50_ levels in the oral rat acute toxicity models have been calculated for further investigation of the in-vivo anticancer activities, and the compounds appear to be quite safe (2.42–2.76 mol/kg)^41^.

According to the previous results, the best-docked molecule **5a** that impact a great inhibition to H-RAS, GTP active form target protein also, showed perfect in-silico physio-chemical, pharmacokinetic and bioavailability without any toxicity and carcinogenicity could be a potential new anticancer drug candidate.

### In vitro studies

#### Growth inhibition of different human cancer cell lines by compound **5a**

**Figure 5:**
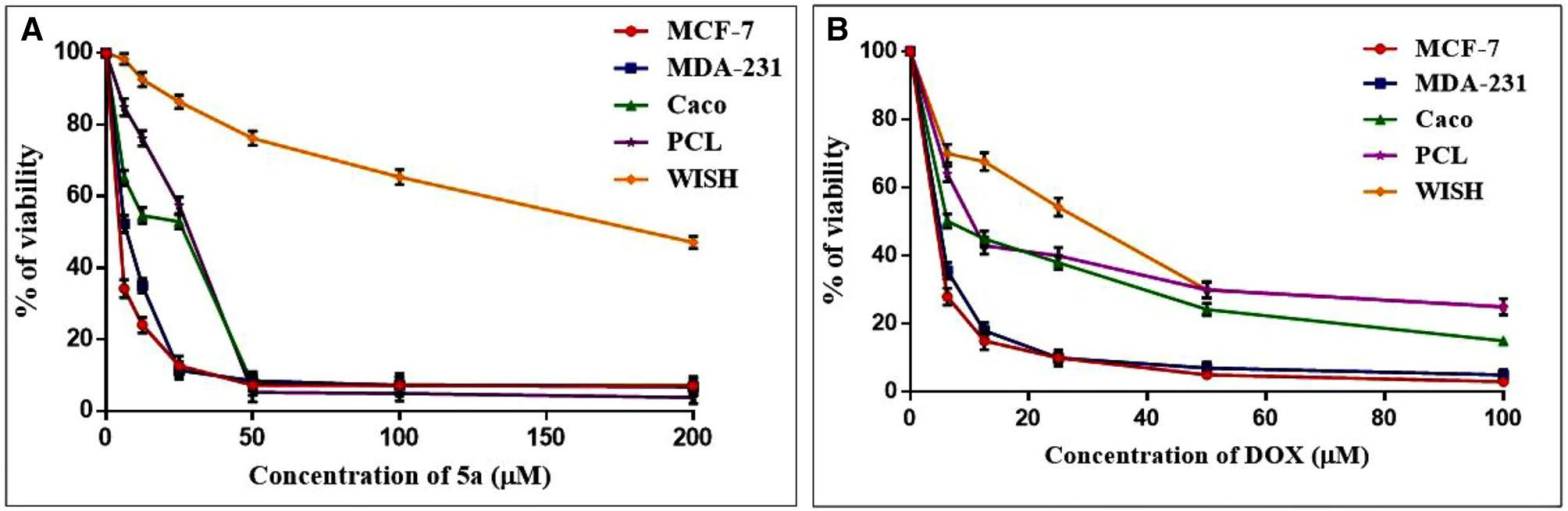
(**A**) Cytotoxicity of **5a** against different cell lines. (**B**) Cytotoxicity of DOX against different cell lines. % Cell viability was plotted against concentrations to determine IC50. The values of IC50 are expressed as mean ± SE of three experiments done in triplets.

In the present study, the cytotoxic effect of the synthesized compound **5a**, and the chemotherapeutic drug, DOX was revealed by MTT assay on different human cancer cell lines including breast (MCF-7 and MDA-MB-231), colorectal (Caco-2), and pancreatic (PANC-1) cancer cells as well as normal cells (WISH). Compound **5a** caused dose-dependent suppression of MCF-7, MDA-MB-231, Caco-2, and PANC-1 cancer cell proliferation with IC_50_ values of 2.617 ± 1.6, 6.778 ± 2.2, 14.8 ± 2.5 and 23.58 ± 2.4 µM respectively (Fig. 5A). According to our finding, compound **5a** exhibited the lowest IC_50_ value against MCF-7 cells indicating that they were more sensitive to this compound than other malignant cell lines compared with the cytotoxic effect of DOX that exhibited an IC_50_ value equal to 2.261 ± 1.4 µM (Fig. 5B). On the other hand, compound **5a** exhibited less cytotoxicity to the normal cell line with an IC_50_ of 180.5 ± 2.7 µM in comparison with cancer cell lines, which proved its safety on normal cells. Contrarily, DOX showed a significant toxic effect against normal cells with an IC_50_ of 24.49 ± 2.1 µM. Therefore, we extended our study using the MCF-7 cells.

#### Alternation of the morphological features of MCF-7 cells by compound **5a**

**Figure 6:**
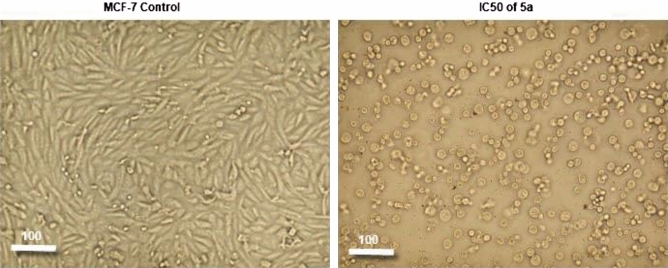
Morphological features of the cell death in MCF-7 cells treated with IC50 of the compound **5a** after 48 h treatment.

In this study, MCF-7 cells treated with an IC_50_ of **5a** showed detectable morphological signs of apoptosis such as cell rounding and shrinkage with decreased cell number detachment, and cytoplasmic condensation. However, the untreated cell morphology appeared normal and confluent. These findings provided more information regarding the capacity of compound **5a** to trigger cell death (Fig. 6).

#### Induction of cell cycle arrest of the MCF-7 cells by compound **5a**

**Figure 7:**
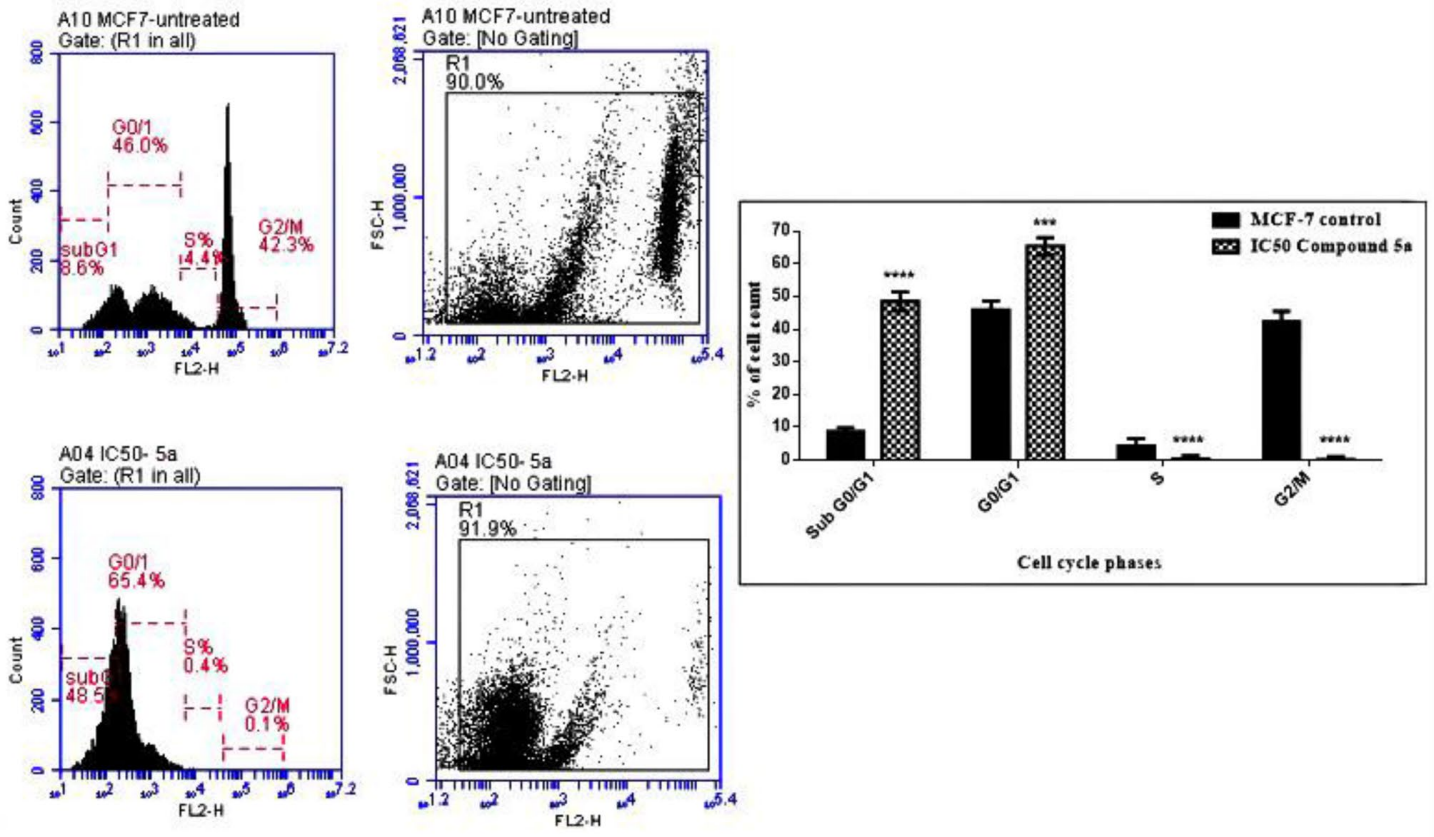
Cell cycle phases in MCF-7 cells treated with IC_50_ of compound **5a** after 48 h treatment.

Cell cycle analysis was conducted on breast cancer cells to better understand the mechanism of the **5a**-mediated tumor growth inhibition, and the findings are shown in Fig. 7. In the MCF-7 untreated cells, oncogenic RAS-induced proliferative signals resulted in the upregulation of transcriptional factors required for cell cycle entrance and cellular progression. Additionally, the metabolic stability of cyclin D1 was affected by the suppression of glycogen synthase kinase (GSK) 3β, which is correlated with continuous ubiquitination and proteasomal degradation of cyclin D1 via PI3K-dependent phosphorylation^17^. Moreover, oncogenic RAS accelerates cell cycle progression by reducing the inhibition caused by antigrowth signaling pathways which inhibit cyclin-dependent kinase (CDK) inhibitors like p21^42^. Oncogenic RAS may contribute to gene instability in cancer via the promotion of G_2_/M phase transition, inhibition of G_2_ DNA checkpoints, and induction of defects in mitotic spindle checkpoints^43^. Herein, by treating the MCF-7 cells with the IC_50_ of compound **5a** the percentage of the cells was significantly arrested at sub-G0/G1 at a rate of 48.5%, compared with that of the untreated control MCF-7 cells (8.6%). These results confirmed the apoptosis of the MCF-7 cells.

#### Blocking of the RAS/PI3K/Akt/JNK signaling pathways in MCF-7 cells by compound **5a**

The elucidation of intracellular signaling pathways and the identification of oncogenes have ushered in a new era of targeted therapy that has greatly improved the prognosis for many malignancies. New therapies targeting these proteins and their downstream mediators have established their capability to stabilize and shrink tumors^44^.

RAS oncogenes are the most frequently mutated oncogenes in human cancer, and RAS-mutant malignancies account for a significant portion of all human diseases^45^. RAS proteins cycle between two conformational states, that is, the active form when they are bound to GTP and the inactive form when bound to GDP. Guanine nucleotide exchange factors (GEFs) are known to stimulate GTP-bound RAS formation, whereas GTPase-activating proteins (GAPs) promote GTP hydrolysis on RAS thereby returning them to the inactive state^46^. In numerous human malignancies, including breast carcinoma, active forms of RAS have been found to induce cellular transformation. Thus, one of the therapeutic approaches to treating breast cancer is the efficient control of oncogenic RAS^47^. In this study, western blot analysis revealed that treatment with the IC_50_ of **5a** significantly decreased the levels of *p*-RAS proteins as compared to the MCF-7 untreated cells. This suggested the antiproliferative nature of this novel compound (Fig. 9).

Activated RAS enhances cellular proliferation by modifying a wide range of intracellular effector pathways that eventually converge to promote growth and suppress apoptotic signals in transformed cells. The PI3K-Akt pathway has been described as a dominant growth survival pathway in breast cancer. The elevation of PI3K-Akt signaling is a common mechanism that malignant cells require to proliferate and escape programmed cell death^48^. Hence, in the present study, we examined the effect of **5a** on the RAS/PI3K/Akt signaling pathway by assessing PI3K, Akt, and p21 gene expression as well as p53 protein expression in MCF-7 cells.

**Figure 8:**
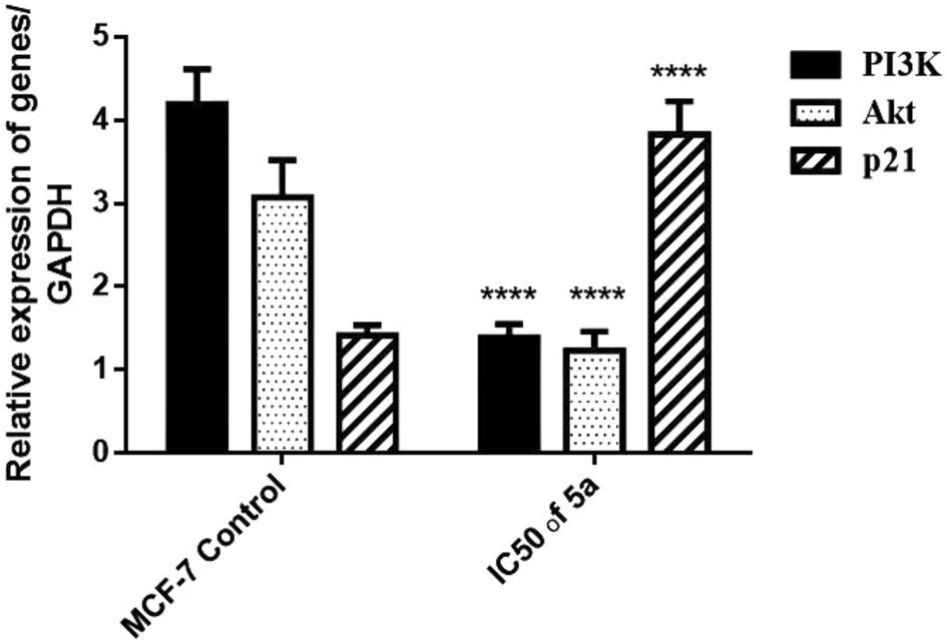
Relative expression of PI3K, Akt, and p21 genes in human breast cancer cells MCF-7 after 48 h treatment. IC_50_ dose of **5a** treatment downregulated PI3K and Akt while upregulated p21 gene expression in MCF-7 cells. Results are expressed as mean ± SE, (n = 3). *p* < 0.05 is considered significant, where *: significantly different from the MCF-7 control group.

RAS protein interacts with PI3K, thereby, resulting in the phosphorylation and activation of Akt^46^. PI3K phosphorylates inositol phospholipids and generates the secondary messenger, phosphatidylinositol-3,4,5-triphosphate (PIP3), which triggers the phosphorylation and activation of the downstream serine/threonine kinase Akt. In turn, Akt promotes cell survival by modulating key proteins with various biological functions^49^. In this study, the IC_50_ of **5a** treatment significantly reduced mRNA transcript level of PI3K and Akt compared with the MCF-7 untreated cells which strongly indicated the inhibitory effect of **5a** on the RAS/PI3K/Akt pathway (Fig. 8).

**Figure 9:**
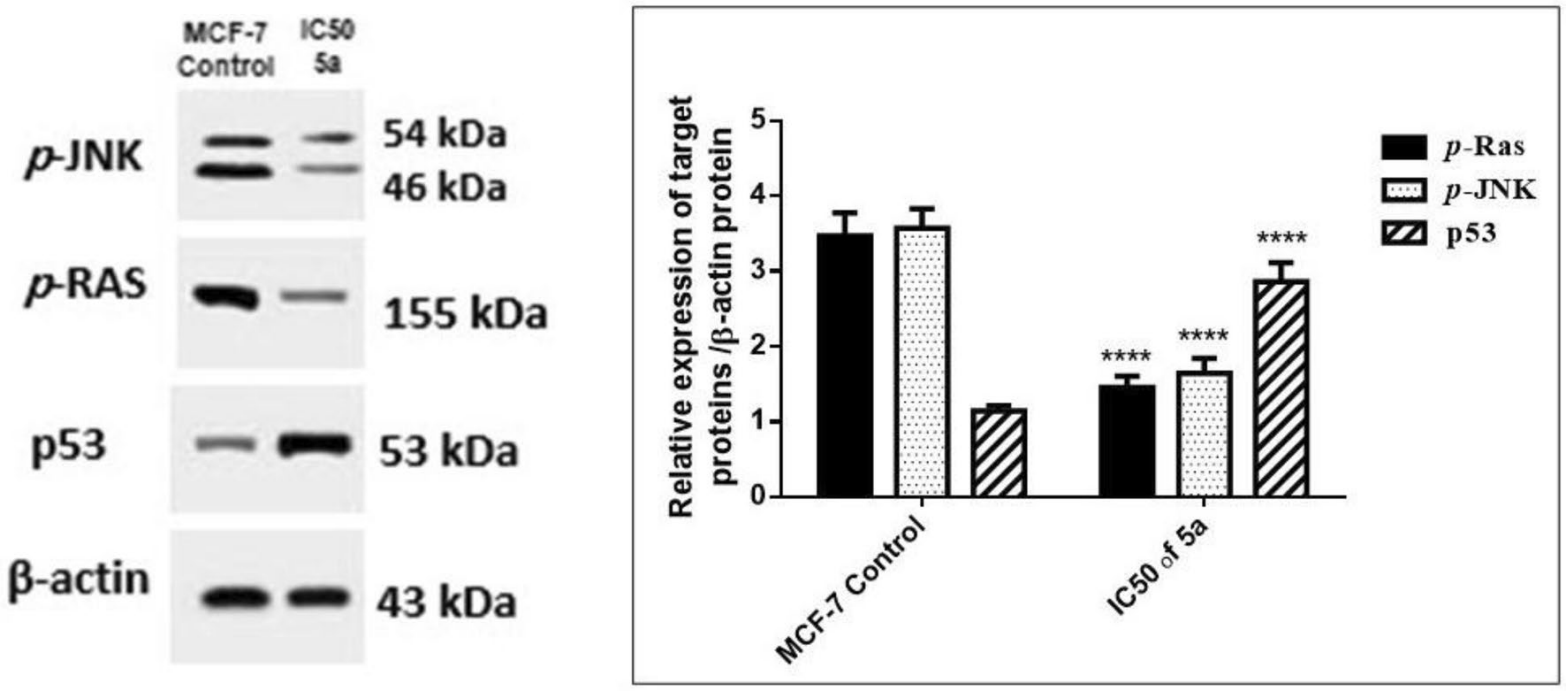
Relative expression of *p*-JNK, *p*-RAS and p53 cropped gel proteins/β-actin protein in human breast cancer cells MCF-7 after 48 h treatment. Results are expressed as mean ± SE, (n = 3). *p* < 0.05 is considered significant, where * : significantly different from the control group. The original blots/gels are presented in supplementary Figs. (S-27 for *p*-JNK, S-28 for *p*-RAS, S-29 for p53, and S-30 for β-actin).

Additionally, phosphorylated Akt is a well-established cell survival factor because it downregulates both p21 and p53^50^. In this work, the treatment with the IC_50_ of **5a** significantly upregulated p21 gene expression compared with the untreated cells (Fig. 8). Moreover, the immunoblot analysis confirmed a significant elevation of p53 protein expression in the **5a**-treated cells compared with that in the MCF-7 untreated cells (Fig. 9). After the treatment with **5a**, the upregulation of p21 gene expression and p53 protein expression confirmed the antineoplastic effect of **5a** toward human breast cancer cells by suppressing the RAS/PI3K/Akt signaling pathway.

Furthermore, RAS is known to activate the JNK/MAP kinase signaling cascade leading to the phosphorylation of c-Jun and augmentation of the activator protein-1 (AP-1) transcriptional activity. Hence, we investigated the effect of **5a** on the cell survival pathway, that is, the RAS/JNK pathway. Our study showed a significant reduction in the p-JNK protein expression when compared with that in the MCF-7 untreated cells suggesting the inhibitory effect of **5a** on the RAS/JNK pathway (Fig. 9). These results may have been obtained because JNK contributes to the RAS-induced estrogen-independent growth of breast cancer cells. Additionally, the JNK pathway is involved in the expression of matrix metalloproteinases (MMPs) that are associated with extracellular matrix degradation and tumor metastasis. Moreover, there is an association between the JNK/c-Jun activation and angiogenesis in invasive breast carcinoma^51^. Indeed, JNK inhibition can cause apoptosis of tumor cells^52^.

#### SAR (structure-anti cancer activity relationship)

**Figure 10:**
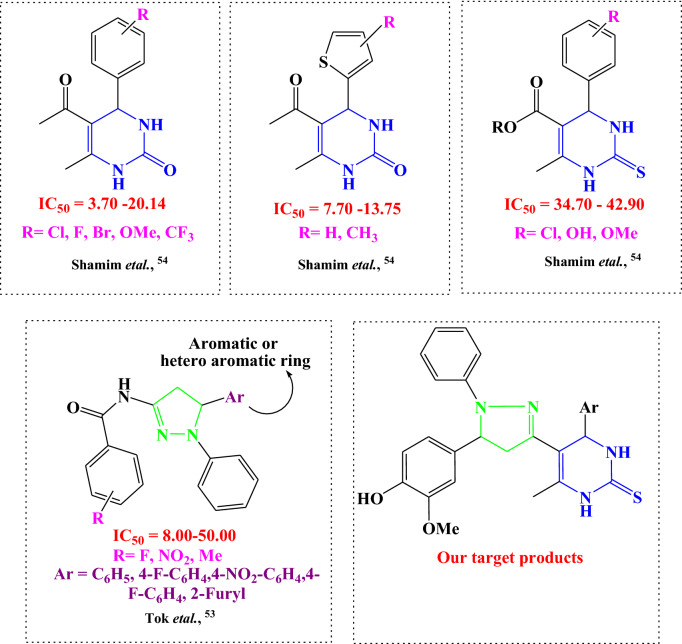
Rational design of newly synthesized pyrimidine-2-thione derivatives.

For novel synthesized compounds, the in vitro anti-cancer and molecular docking results showed the following structure activity relationship (Fig. 10):

- The presence of a bioactive pyrazole ring increases the anticancer activities^53^.
- (–OH) and (-OCH_3_) that was found at the aromatic ring improved the anticancer activities.
- The presence of pyrimidine ring is considered an essential moiety for enhancing anticancer activity^54^.
- The high aromaticity of the prepared compounds enhanced the anticancer activities.

## Conclusion

**Figure 11:**
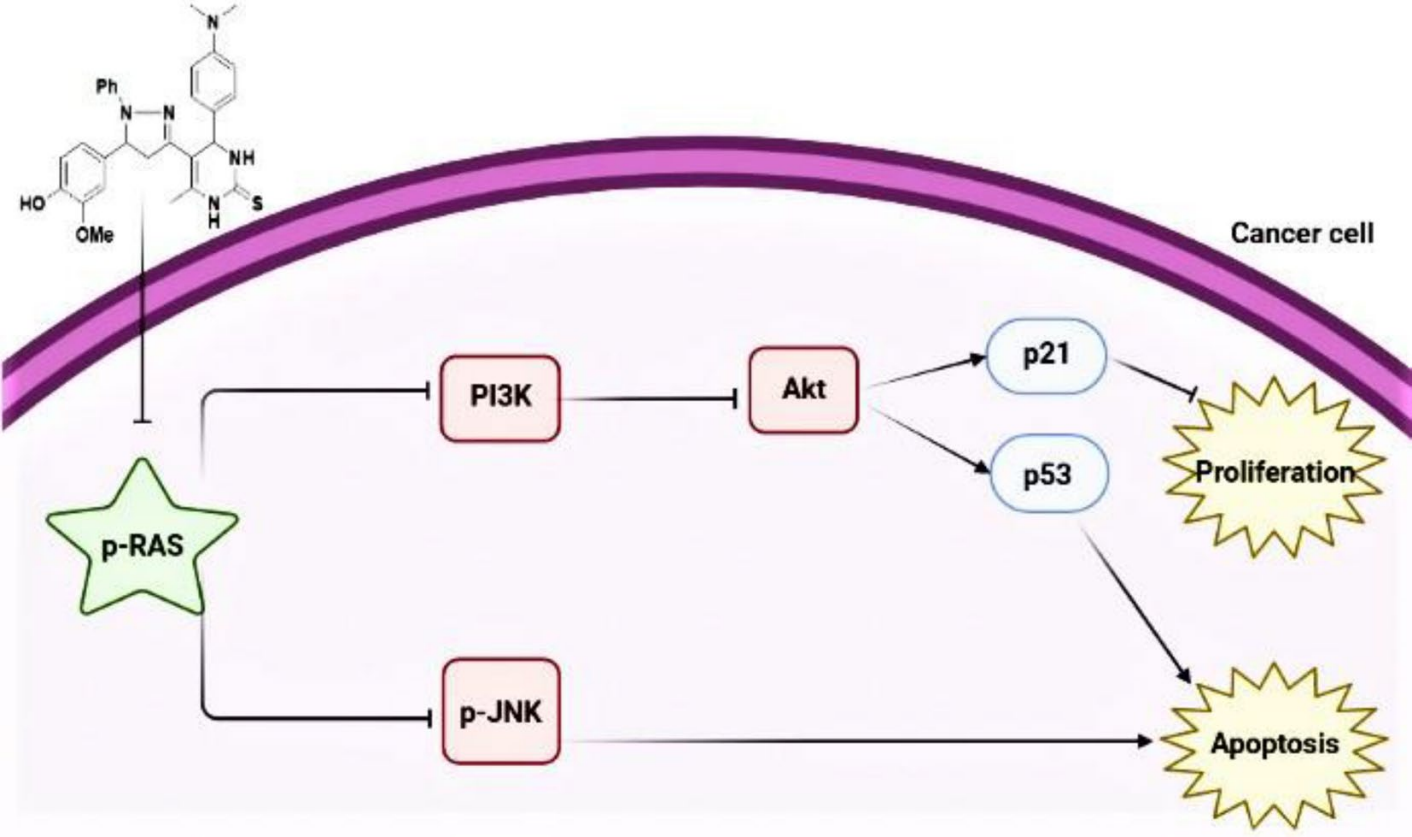
Systematic design for the effect of compound **5a** as inhibitor of breast carcinoma cell proliferation.

Pyrimidine-2-thione derivatives **2**–**6** were successfully synthesized via conventional and ultrasonic irradiation techniques. The ultrasonic technique was found to be more effective than conventional heating, as evidenced by its higher yields and short reaction times. compound **5a** was selected among the others according to their in silico molecular free binding energy docking studies towards inhibiting the H-RAS target protein and obeyed Lipinski’s rule of five. Additionally, the results of the in vitro studies emphasized that the newly synthesized compound **5a** induced cell death via targeting the RAS/PI3K/Akt signaling pathway. Moreover, it upregulated p21 and p53 proteins in the human breast cancer cells. Finally, compound **5a** exhibited antineoplastic activity via the inhibition of *p*-JNK protein in cancer cells. Therefore, **5a** appears to be an attractive antitumor compound with future clinical applications for breast cancer treatment (Fig. 11).

## Supplementary Information

Supplementary Figures.

## Data Availability

The datasets generated and/or analysed during the current study are available in: Macromolecule protein structure, can be deposited in the worldwide protein data bank repository, (https://www.rcsb.org/structure/5P21). mRNA sequences, can be deposited in the National Center for Biotechnology (NCBI), (NM_181524.2**,** XM_047431075.1**,** NM_001374511.1 and NM_001357943.2). All cell lines, were purchased from the American Type Culture Collection (ATCC) organization (#ATCC HTB-22**,** #ATCC HTB-26, # ATCC HTB-37, # ATCC CRL-1469 and # ATCC CCL-25). All primary antibodies for western blot, were purchased from cell signaling, (#3321, #9251, #9282 and #8457).
