# Supplementary material for: Synthesis, molecular docking, and in-vitro studies of pyrimidine-2-thione derivatives as antineoplastic agents via potential RAS/PI3K/Akt/JNK inhibition in breast carcinoma cells
Source: Sci Rep. 2022 Dec 22;12:22146. doi: 10.1038/s41598-022-26571-7 (PMC9780203; doi:10.1038/s41598-022-26571-7)
Supplement: Supplementary file 1 — Supplementary Figures. [file 41598_2022_26571_MOESM1_ESM.pdf]

Supporting Information

Table of Contents

**Name**	**Pages**
**NMR Charts**	**S-2**
**Raw data of docking**	**S-18**
**Docking simulations of all novel compounds using MOE software**	**S-19**
**Bioavailability data of all compounds**	**S-23**
**Raw western blot gel of *p*-JNK**<br>Whole mount and the cropped part of western blot.	**S-26**
**Raw western blot gel of *p*-RAS**<br>Whole mount and the cropped part of western blot.	**S-27**
**Raw western blot gel of p53**<br>Whole mount and the cropped part of western blot.	**S-28**
**Raw western blot gel of *β*-actin**<br>Whole mount and the cropped part of western blot.	**S-29**
**General information**	**S-30**

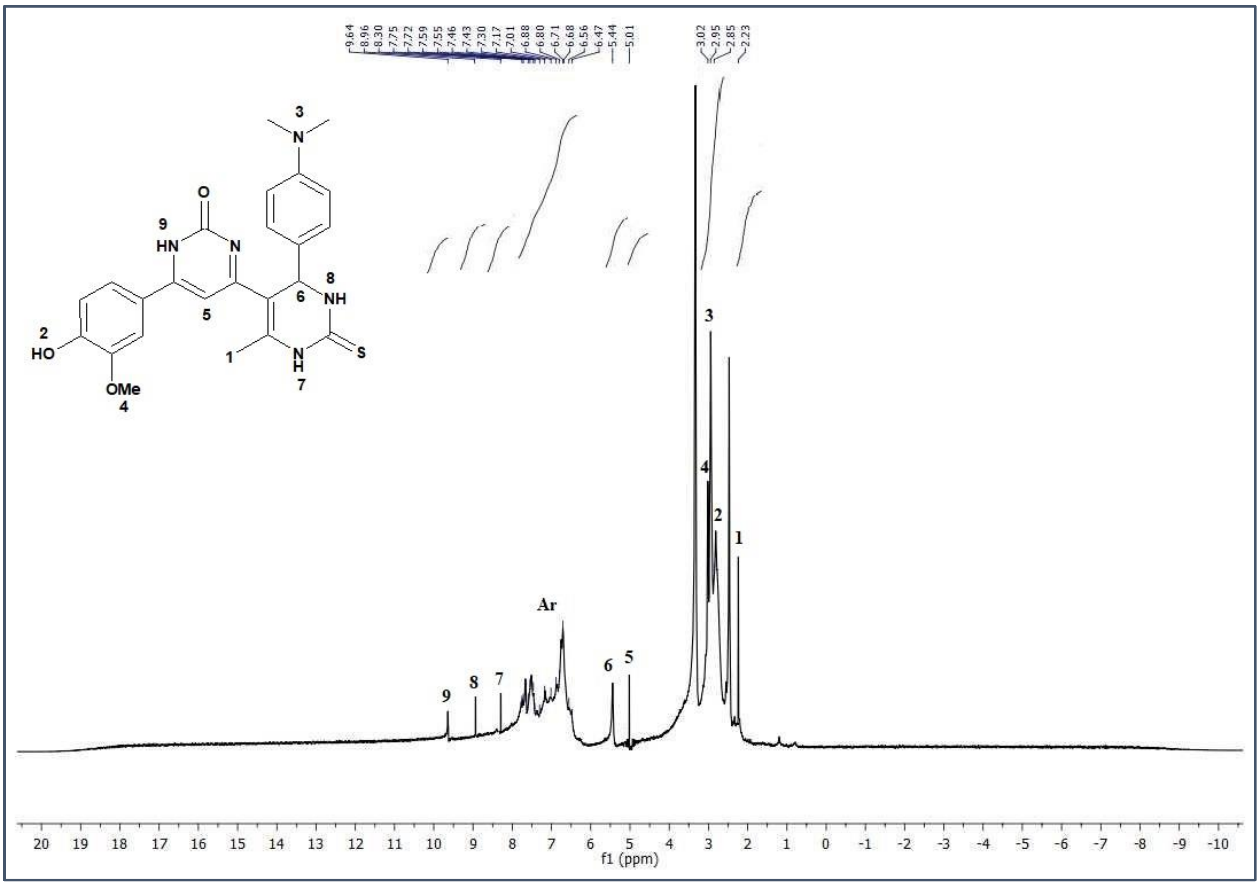

Figure S1 :^{1}H-NMR of compound 3a

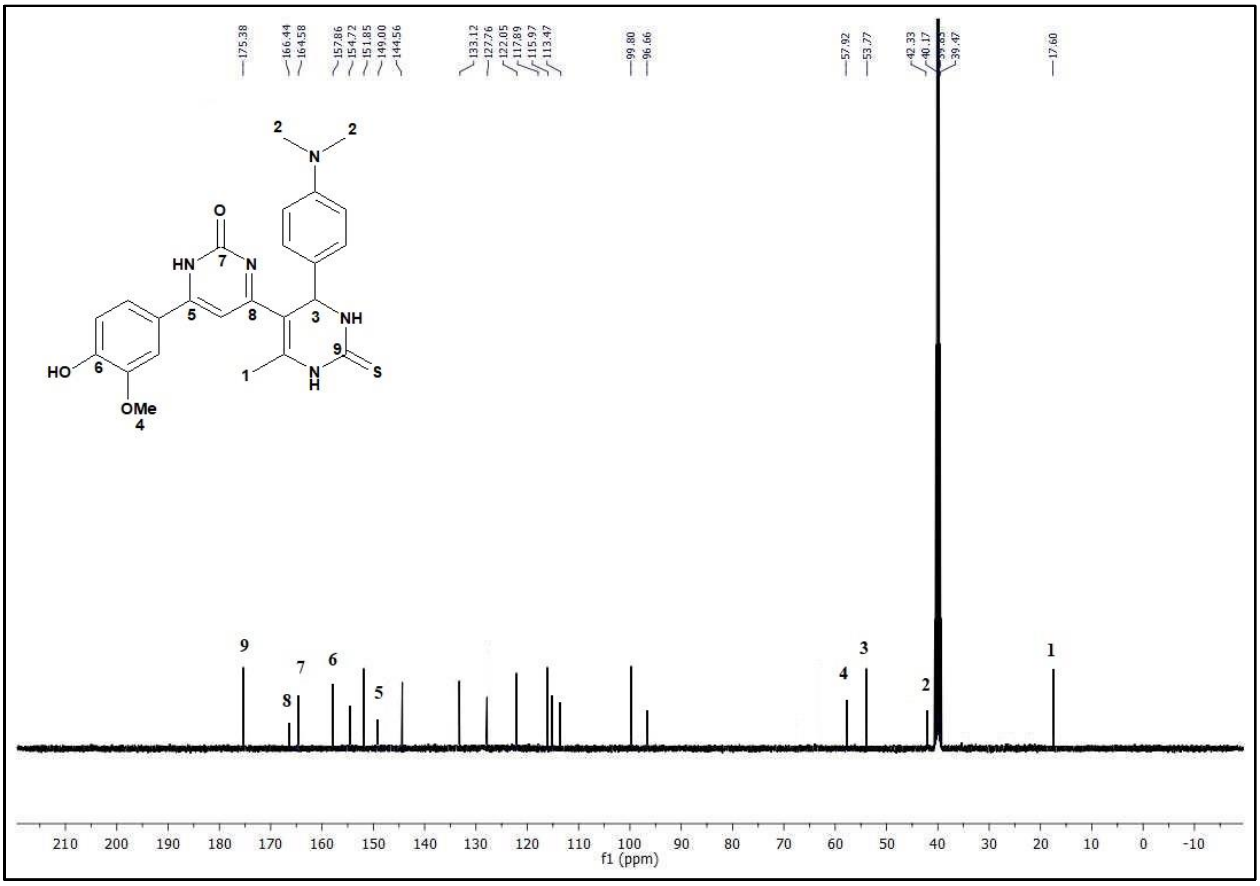

Figure S2 :^{13}C-NMR of compound 3a

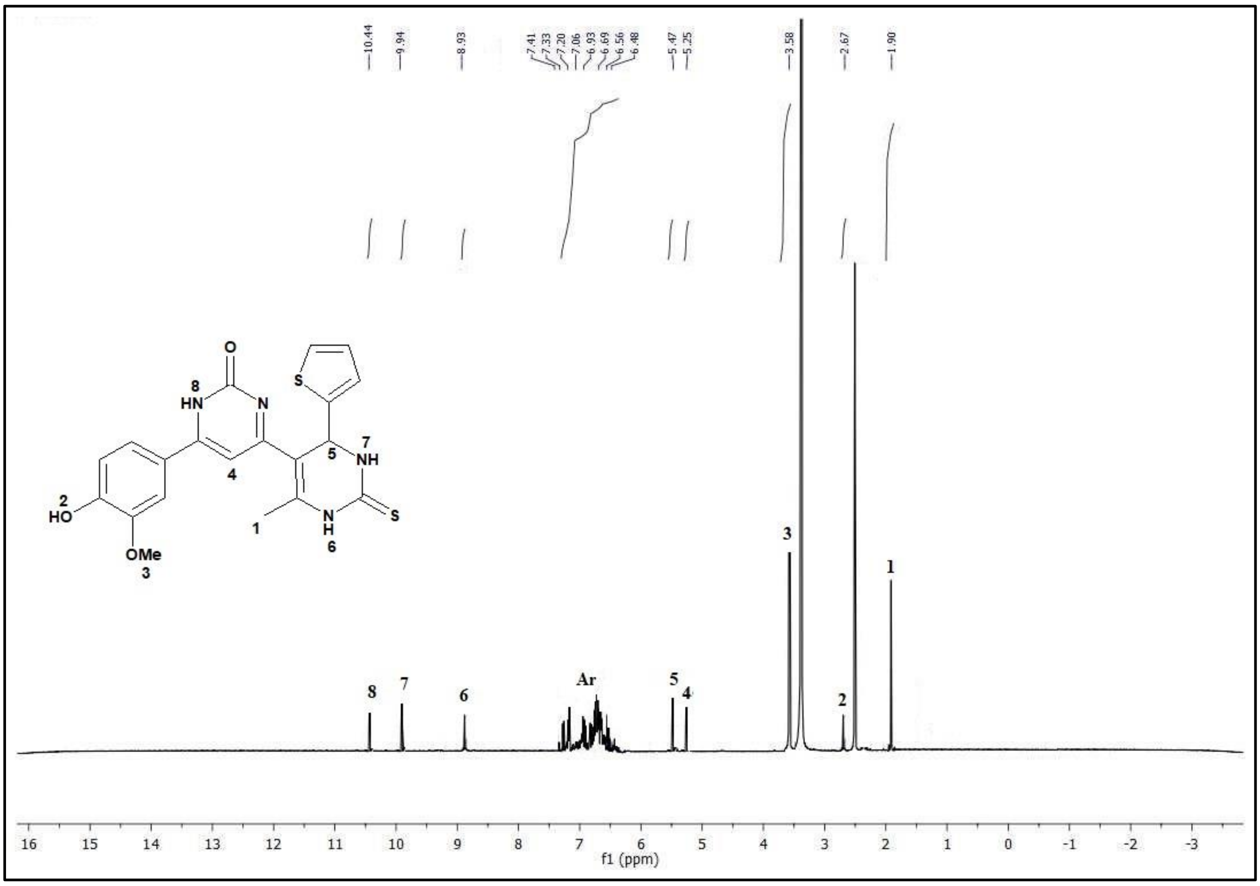

Figure S3 :^{1}H-NMR of compound 3b

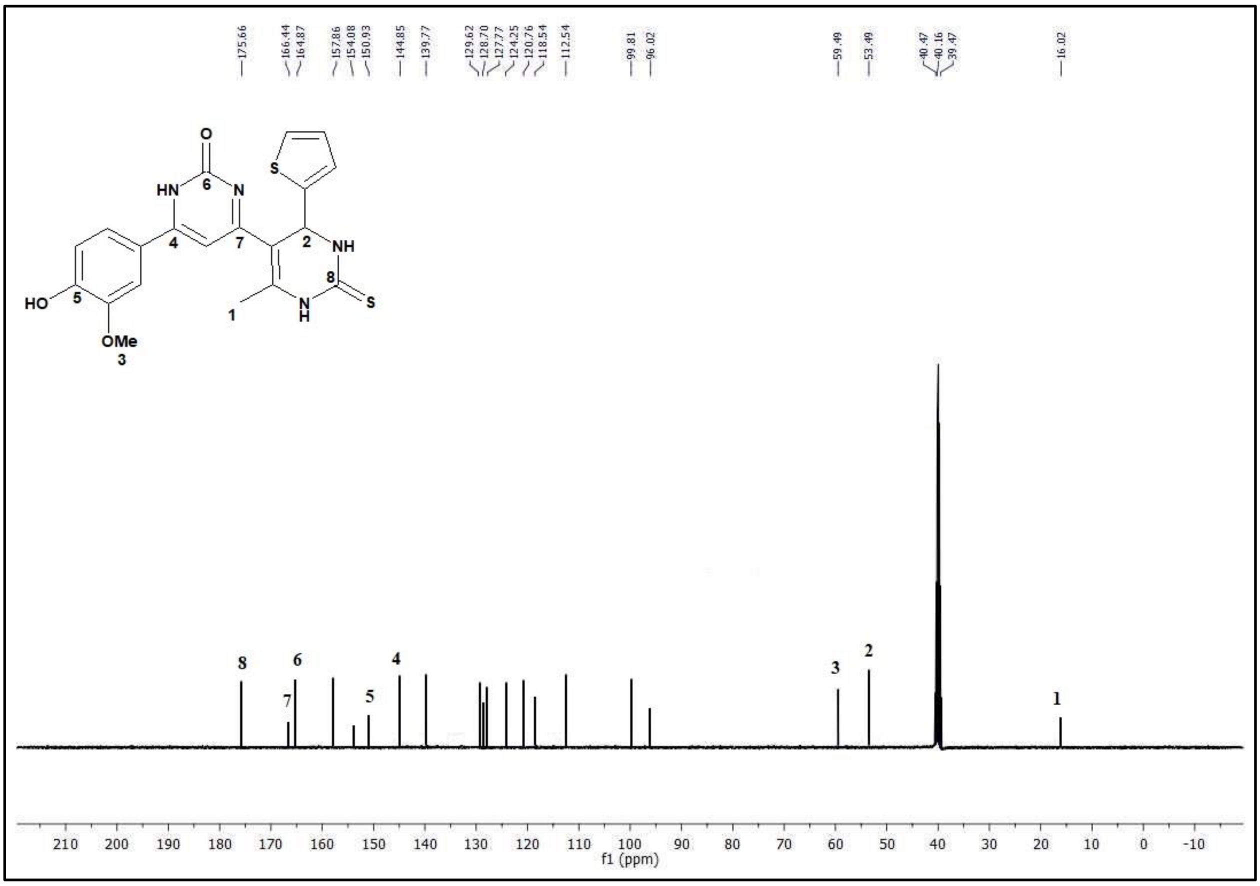

Figure S4 :^{13}C-NMR of compound 3b

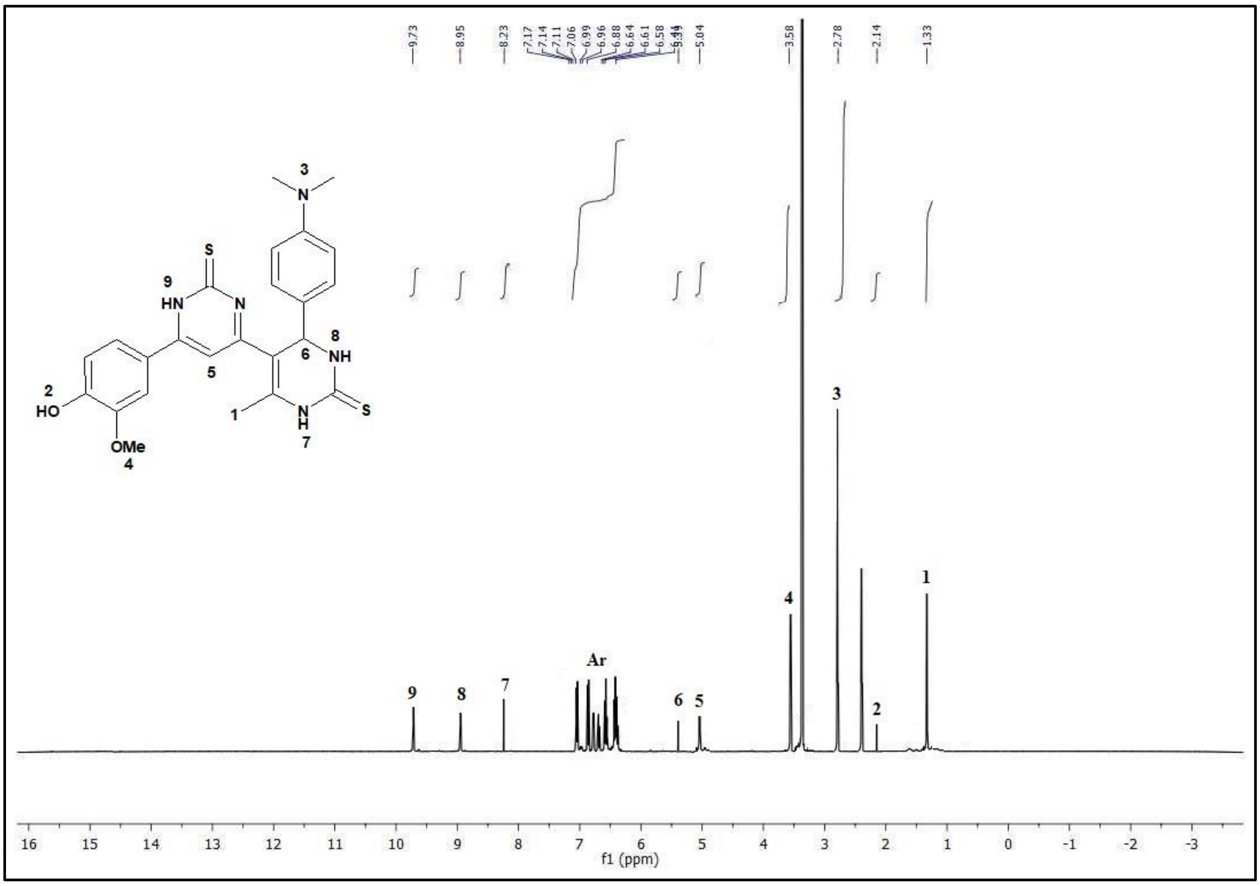

Figure S5 :^{1}H-NMR of compound 4a

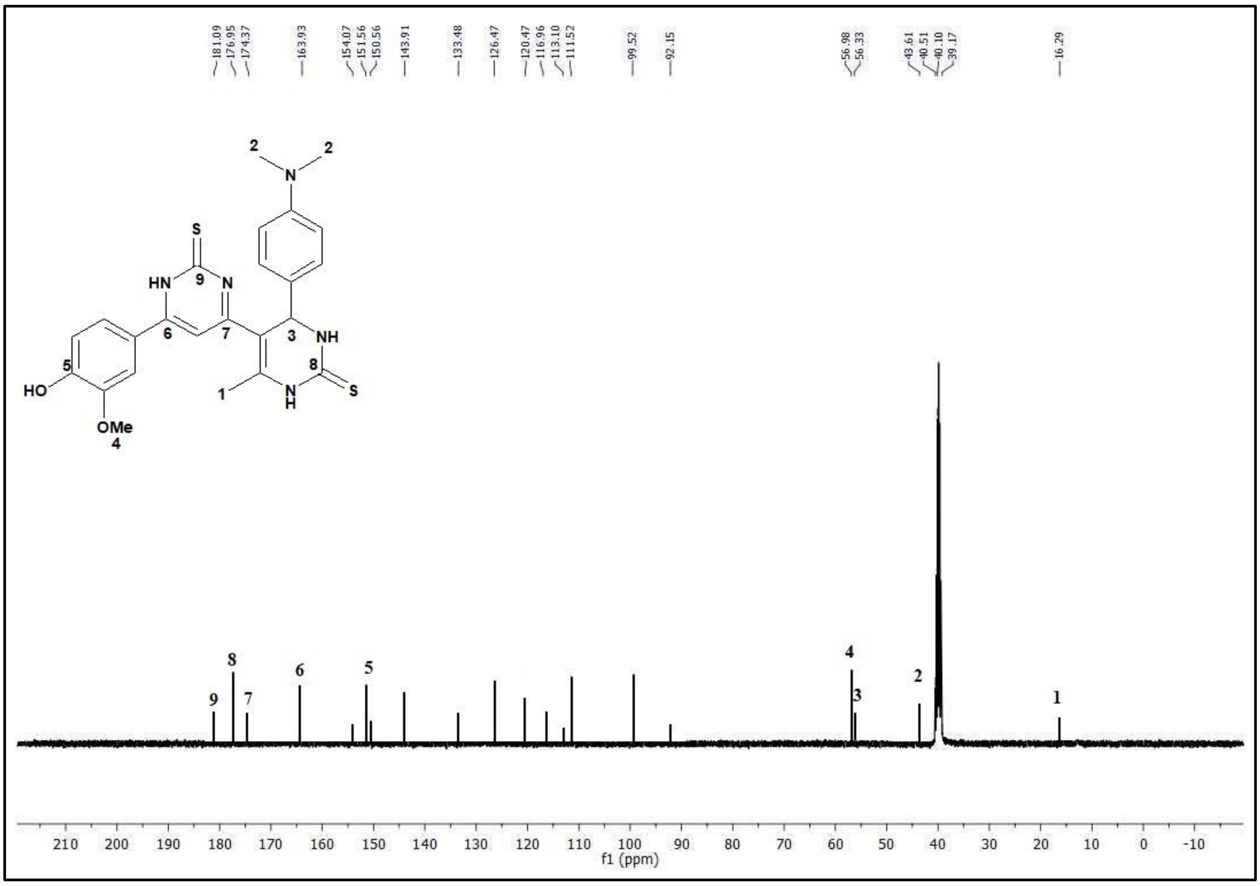

Figure S6 :^{13}C-NMR of compound 4a

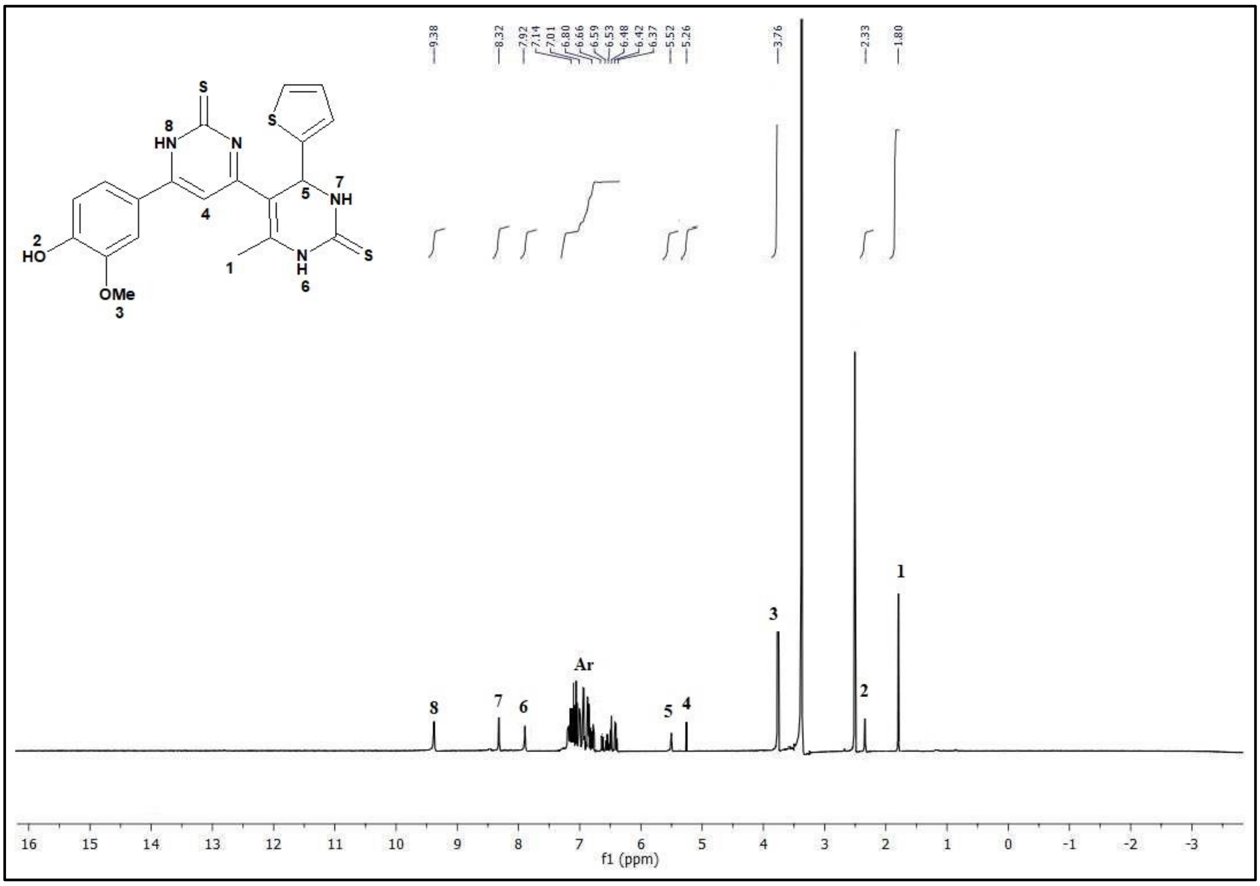

Figure S7 :^{1}H-NMR of compound 4b

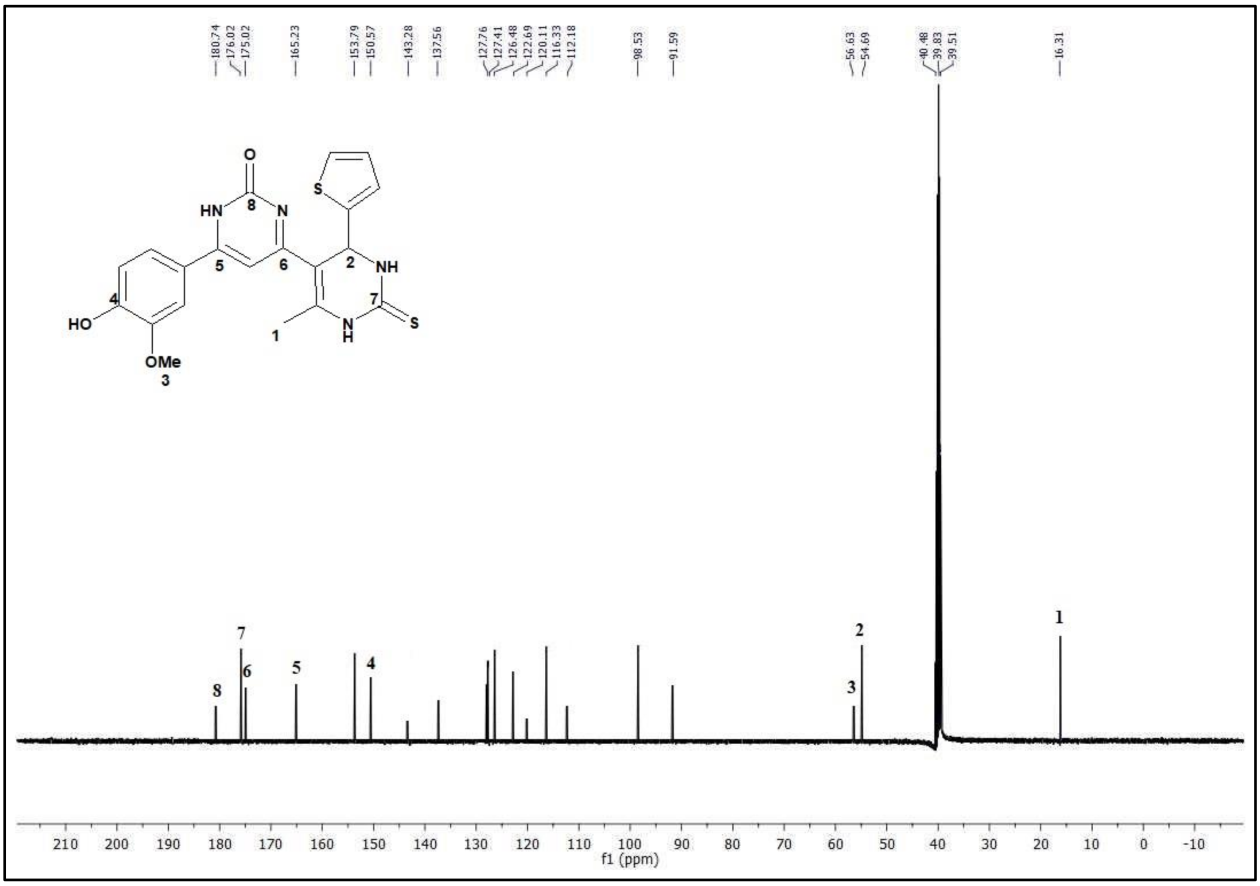

Figure S8 :^{13}C-NMR of compound 4b

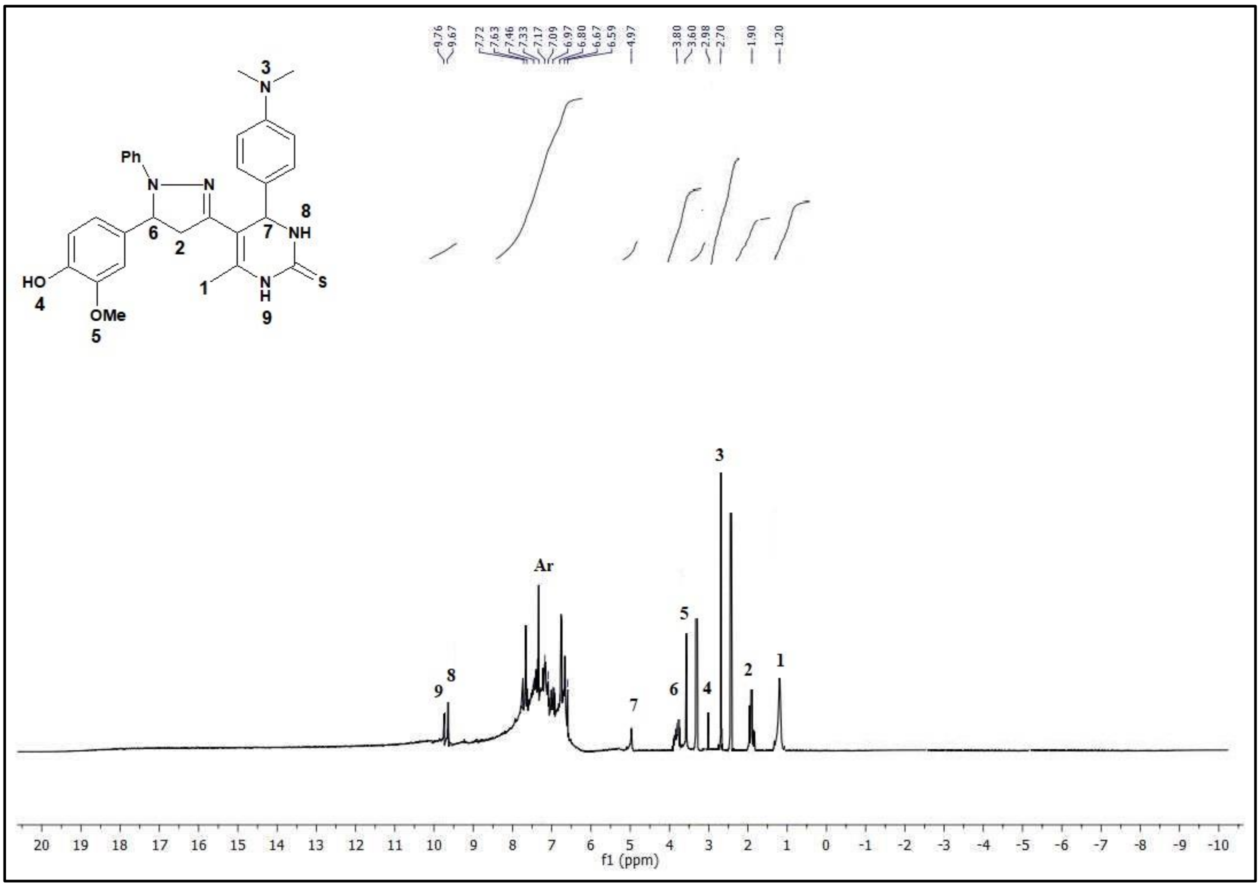

Figure S9 :^{1}H-NMR of compound 5a

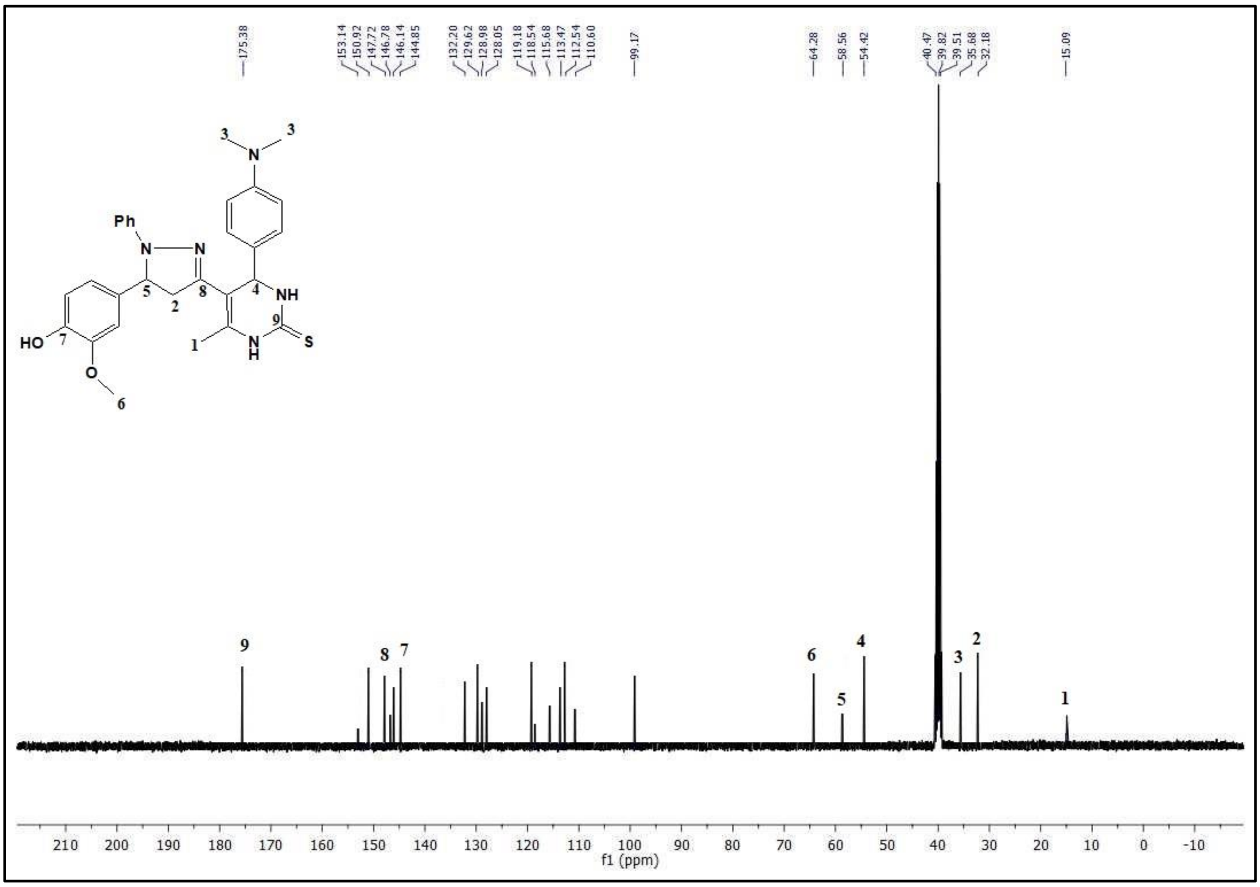

Figure S10 : ^{13}C-NMR of compound 5a

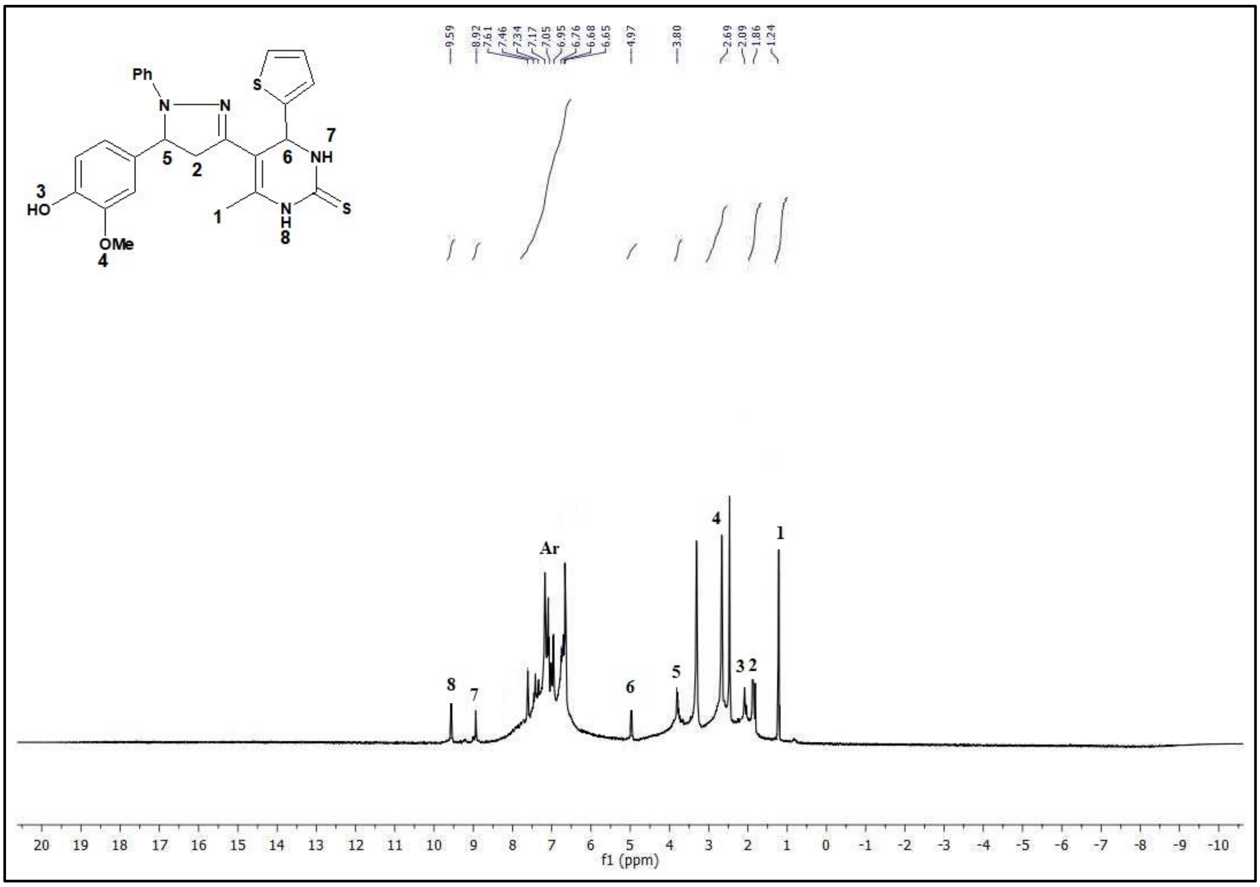

Figure S11 :^{1}H-NMR of compound 5b

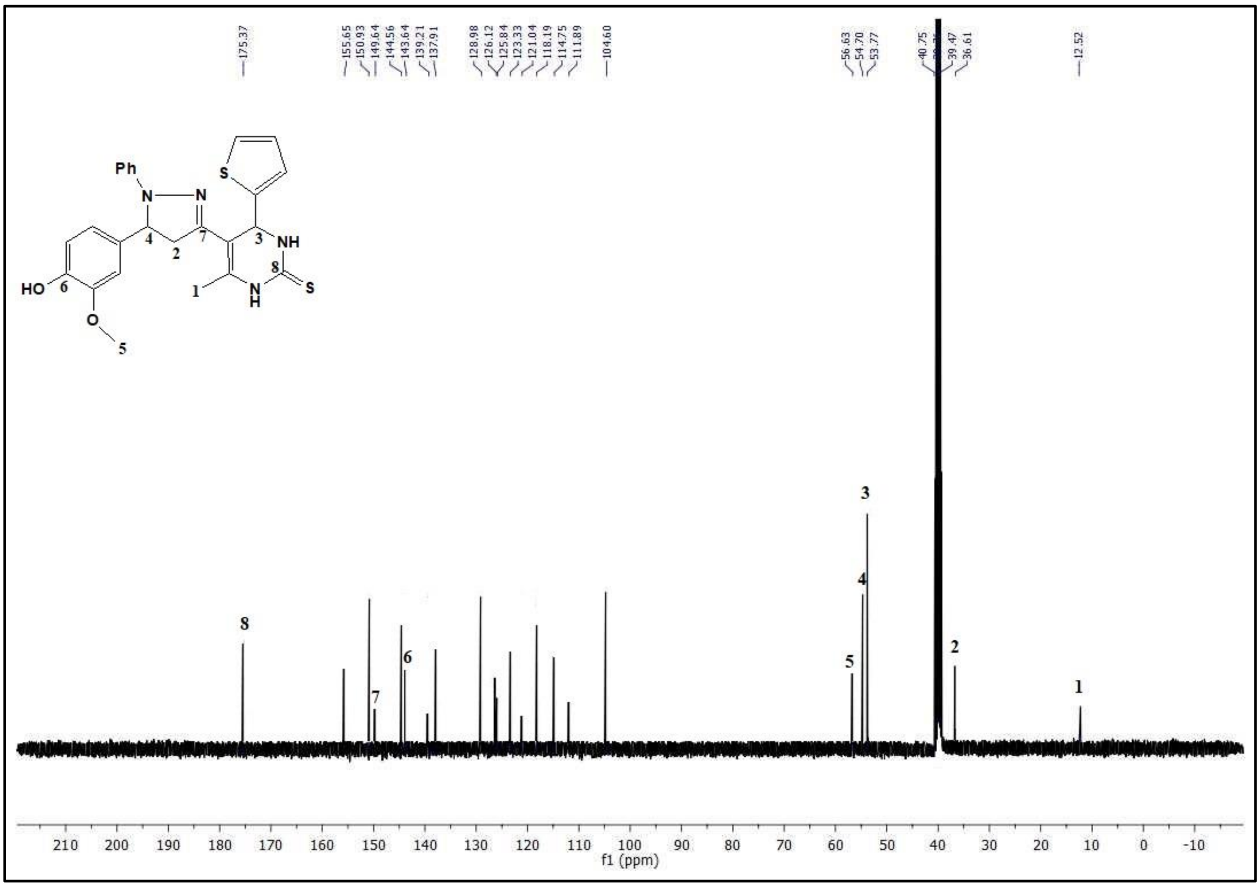

Figure S12 :^{13}C-NMR of compound 5b

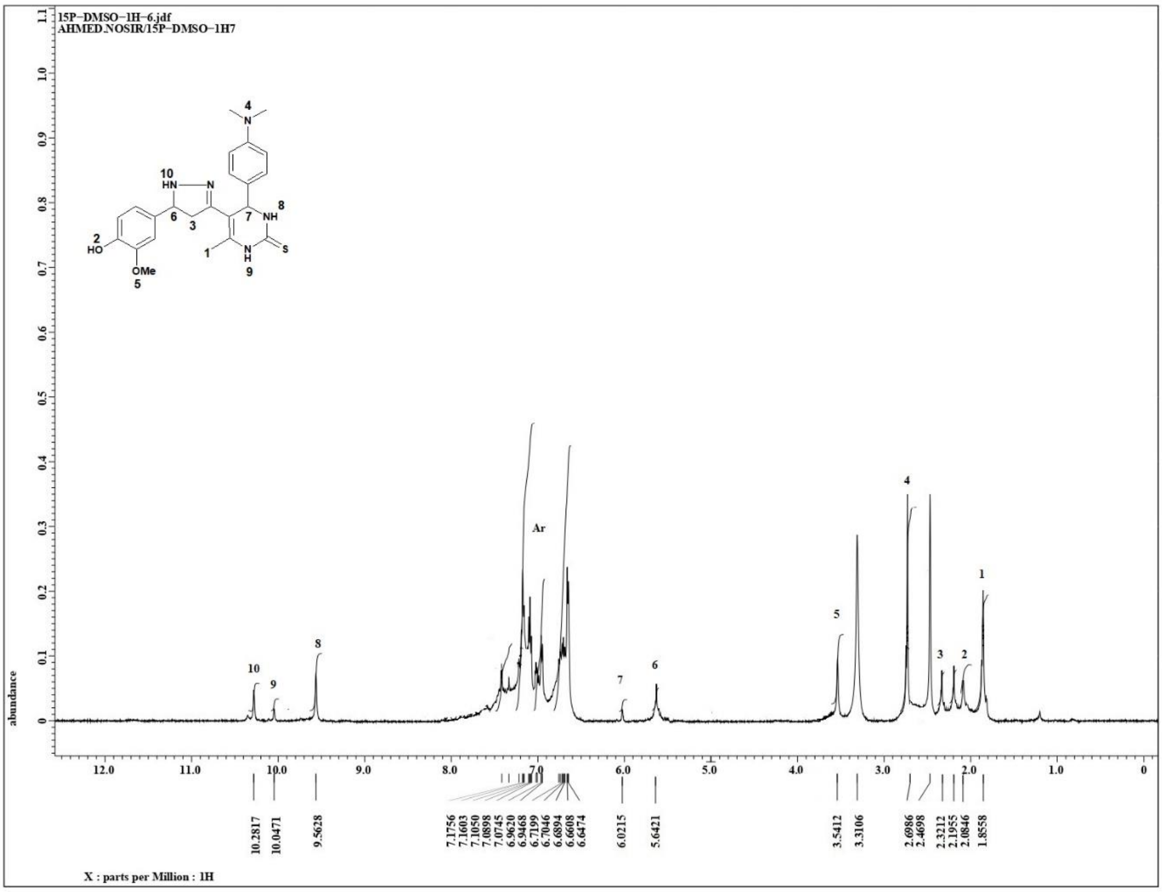

Figure S13 :^{1}H-NMR of compound 6a

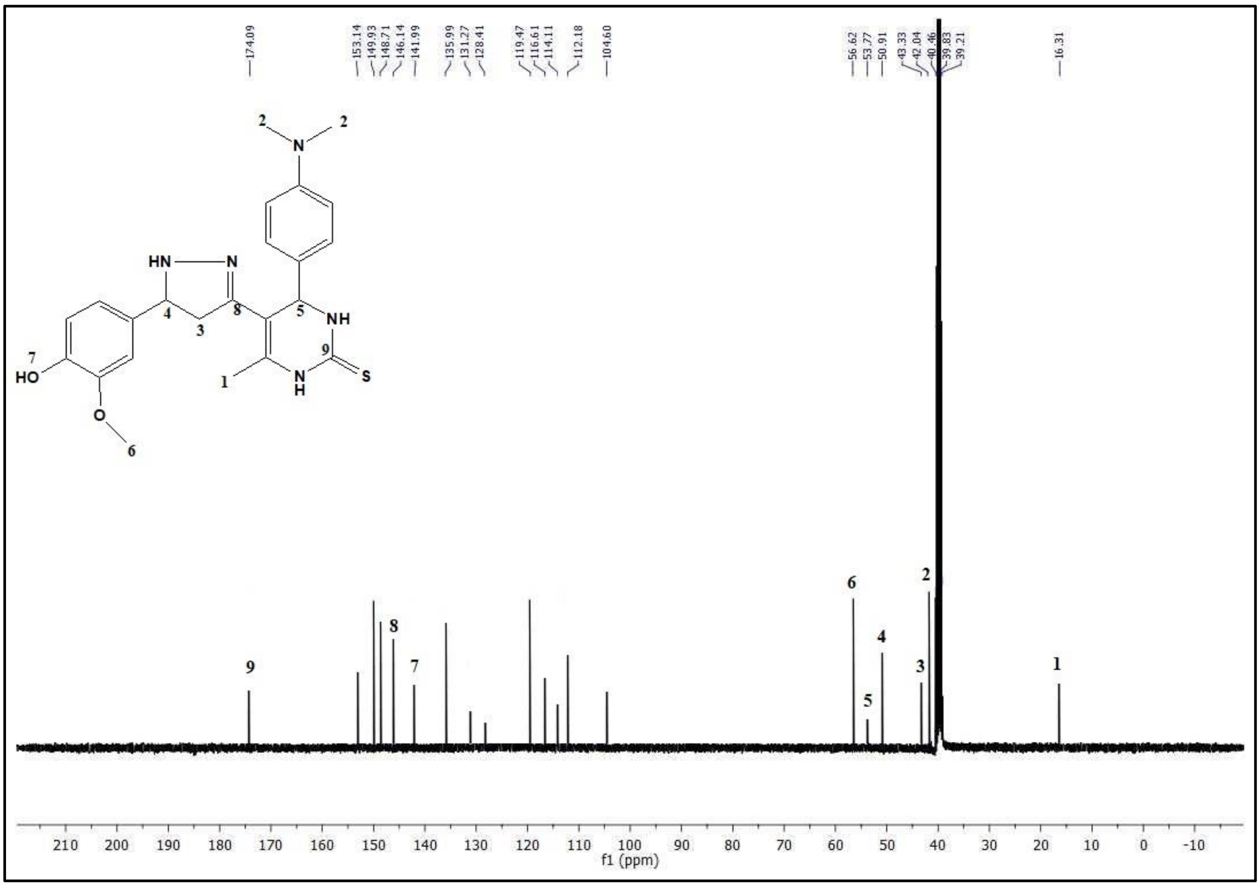

Figure S14 :^{13}C-NMR of compound 6a

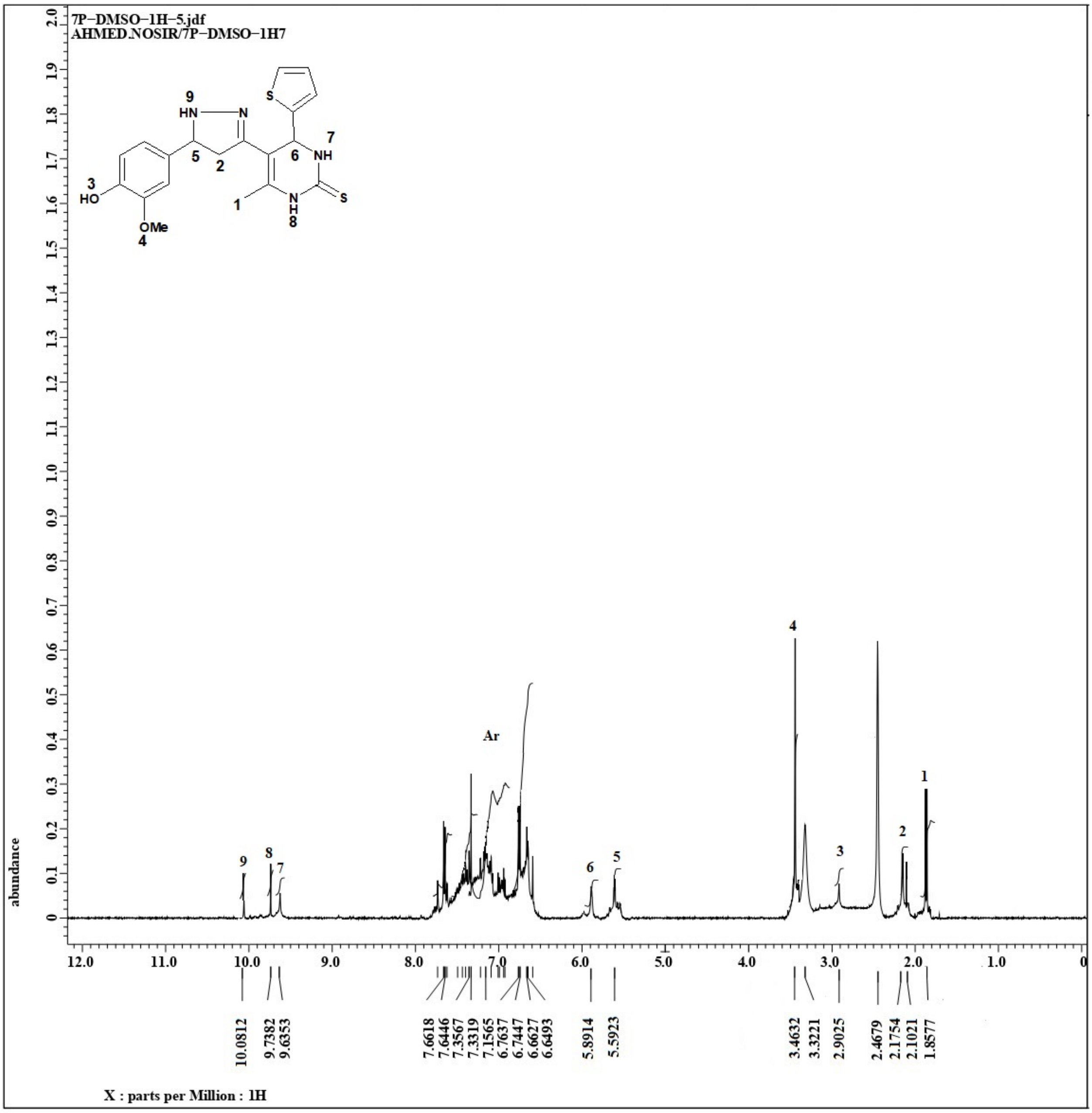

Figure S15 :^{1}H-NMR of compound 6b

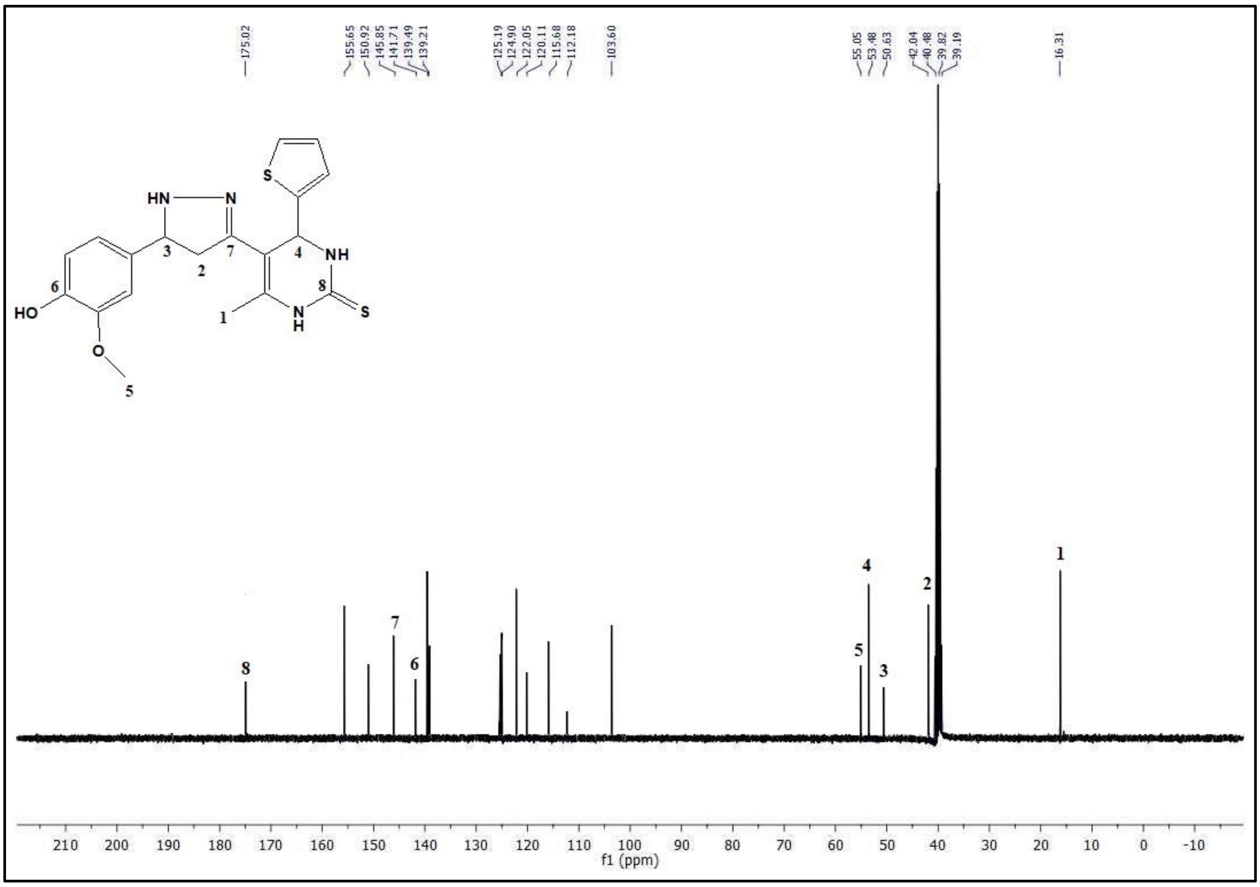

Figure S16 :^{13}C-NMR of compound 6b

Database Viewer : e:/.../moe results of 3a/results - copy.mdb

File Edit Display Compute Window Help — SVL DBV MOE Cancel

	mol	rseq	mseq	Docking score	rmsd_refine	E_conf
1		1	1	-7.9047	2.4456	-155.0565

Raw docking data of 3a

Database Viewer : e:/moe docking/.../results of 3b-copy.mdb

File Edit Display Compute Window Help — SVL DBV MOE Cancel

	mol	rseq	mseq	Docking sc...	rmsd_refine	E_conf
1		1	1	-5.1363	0.9529	-156.4687

Raw docking data of 3b

Database Viewer : e:/moe docking/.../results of 4a - copy.mdb

File Edit Display Compute Window Help — SVL DBV MOE Cancel

	mol	rseq	mseq	Docking sc...	rmsd_refine	E_conf
1		1	1	-8.5877	2.3940	-148.8494

Raw docking data of 4a

Database Viewer : e:/moe docking/.../results of docking 4b - copy.mdb

File Edit Display Compute Window Help — SVL DBV MOE Cancel

	mol	rseq	mseq	Docking sc...	rmsd_refine	E_conf
1		1	1	-7.2252	0.9368	-135.6863

Raw docking data of 4b

Database Viewer : e:/moe docking/.../results of 5a - copy.mdb

File Edit Display Compute Window Help — SVL DBV MOE Cancel

	mol	rseq	mseq	Docking score	rmsd_refine	E_conf
1		1	1	-10.9329	1.9668	-68.9563

Raw docking data of 5a

Database Viewer : e:/moe docking/.../results of 5b - copy.mdb

File Edit Display Compute Window Help — SVL DBV MOE Cancel

	mol	rseq	mseq	Docking sc...	rmsd_refine	E_conf
1		1	1	-6.6447	1.4340	-55.1105

Raw docking data of 5b

Database Viewer : e:/moe docking/.../results of 6a - copy.mdb

File Edit Display Compute Window Help — SVL DBV MOE Cancel

	mol	rseq	mseq	Docking sc...	rmsd_refine	E_conf
1		1	1	-9.4097	1.4156	-76.9313

Raw docking data of 6a

Database Viewer : e:/.../moe results of 6b/results - copy.mdb

File Edit Display Compute Window Help — SVL DBV MOE Cancel

	mol	rseq	mseq	Docking sc...	rmsd_refine	E_conf
1		1	1	-6.9546	1.2267	-65.8531

Raw docking data of 6b

rseq: The receptor sequence number; **mseq**: The molecule sequence number.; **rmsd_refine**: The root square deviation between the pose before refinement and the pose after refinement; **E_conf**: The energy of the conformer; **Docking score**: The final score, which is the score of the last stage.

Figure S17: Raw docking data of compounds 3-6

3a + H-RAS, GTP active form

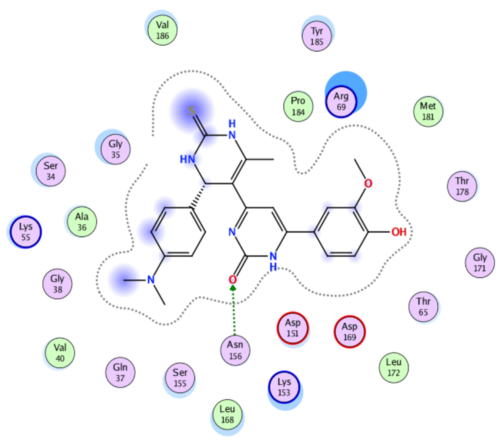

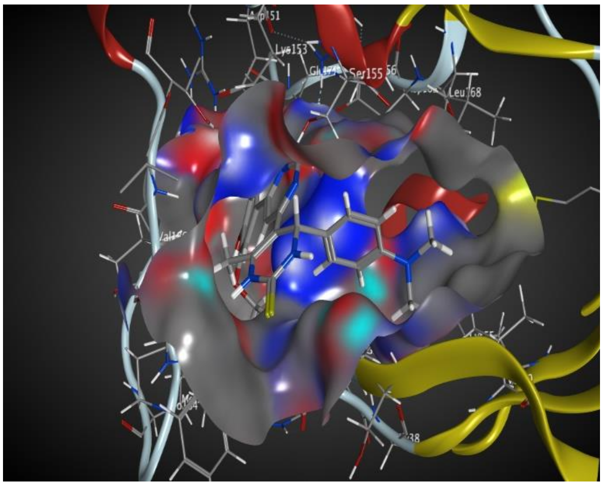

3b + H-RAS, GTP active form

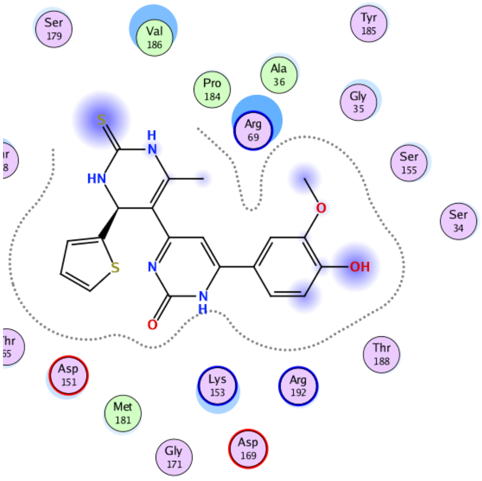

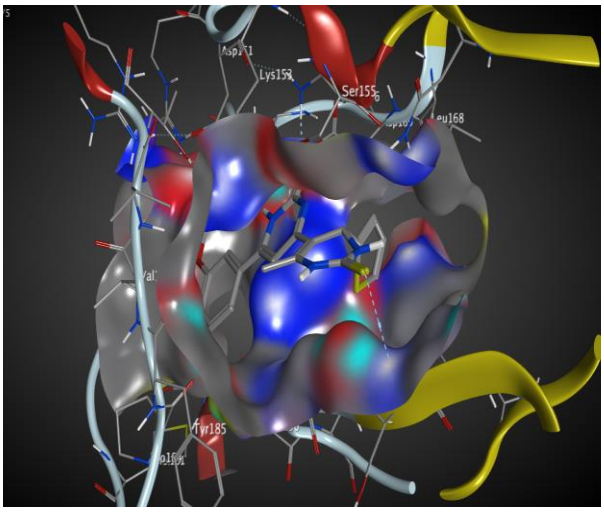

Continued overleaf

4a + H-RAS, GTP active form

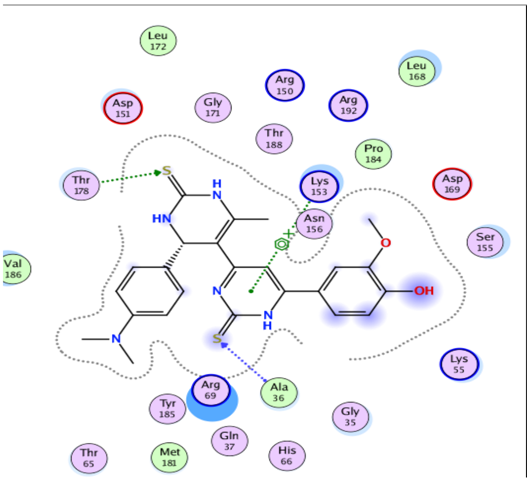

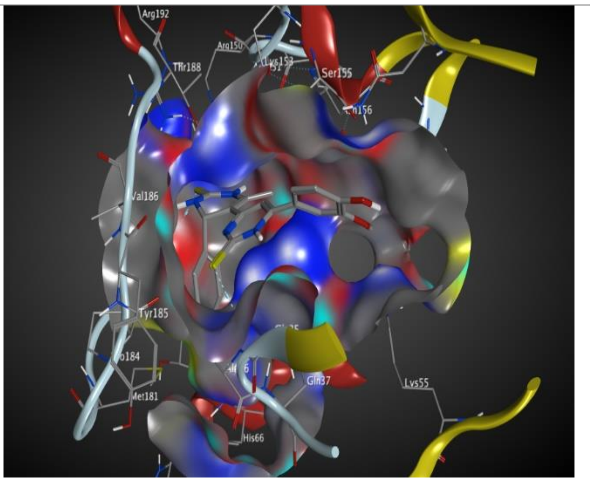

4b+ H-RAS, GTP active form

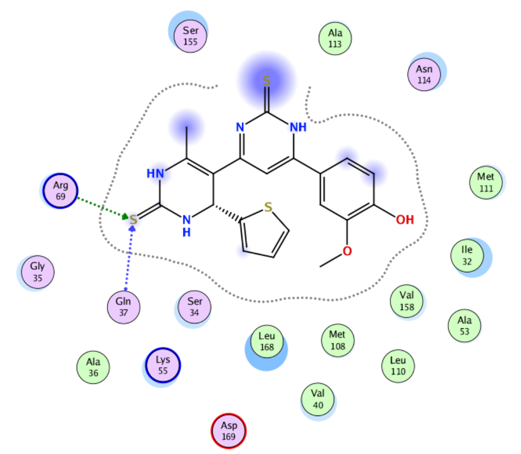

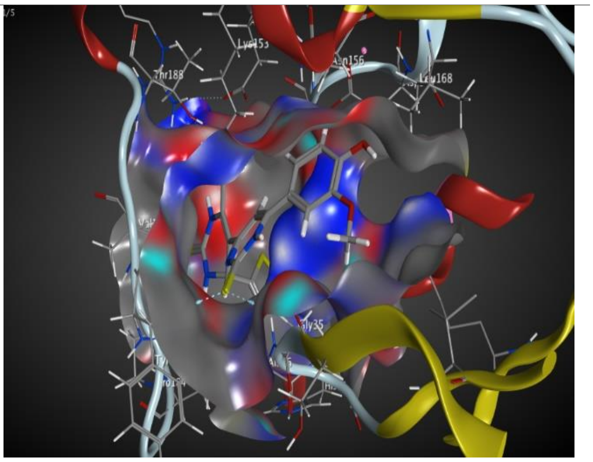

Continued overleaf

5a+ H-RAS, GTP active form

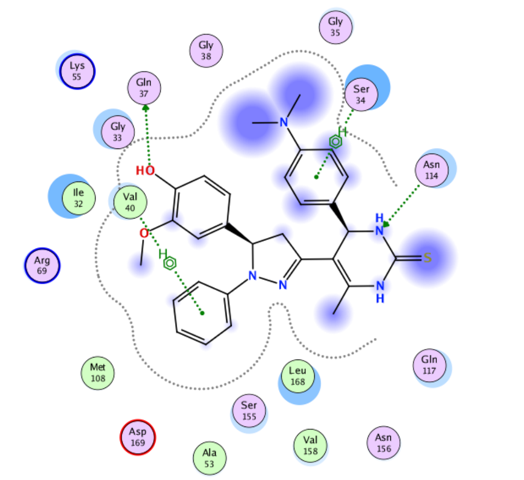

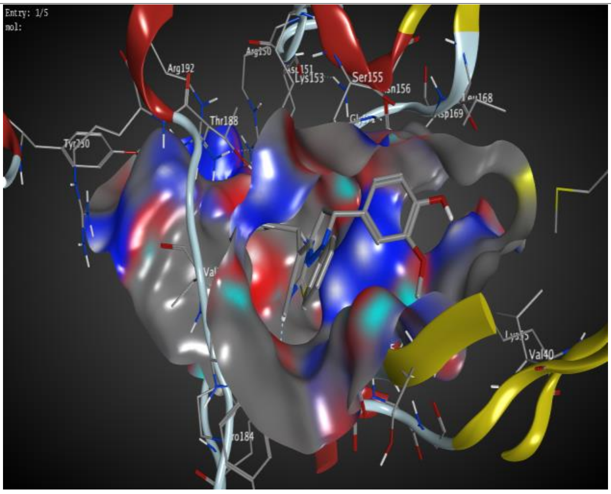

5b+ H-RAS, GTP active form

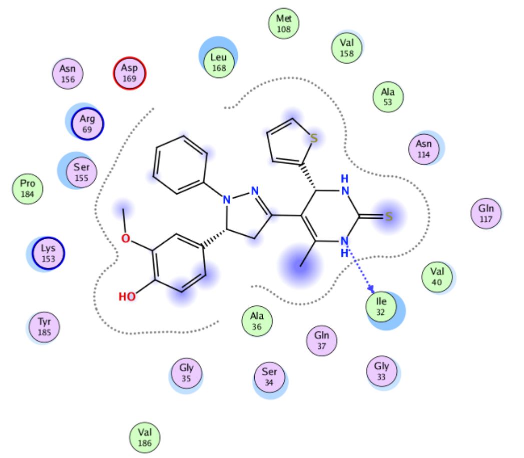

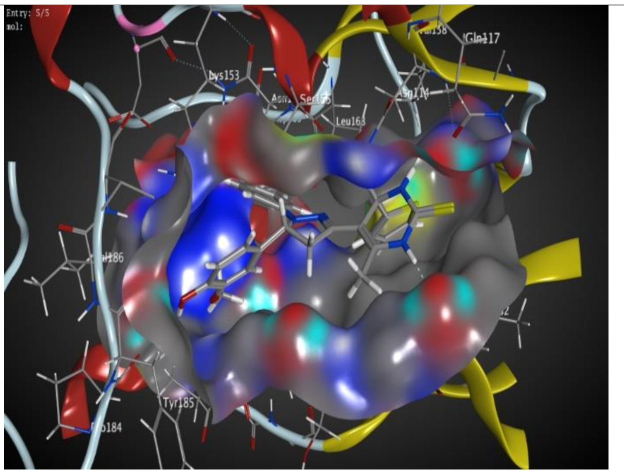

Continued overleaf

6a+ H-RAS, GTP active form

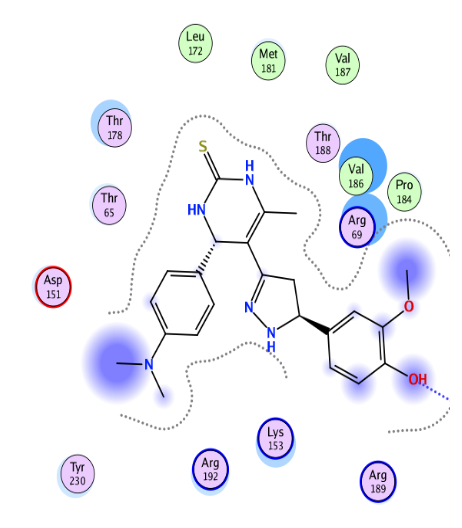

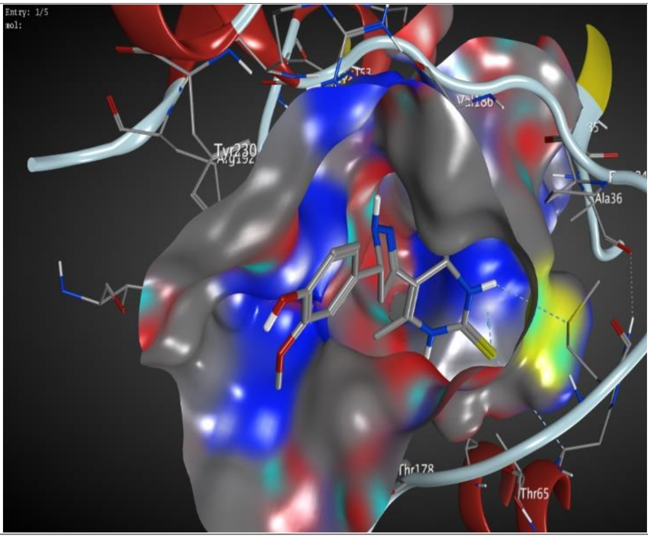

6b+ H-RAS, GTP active form

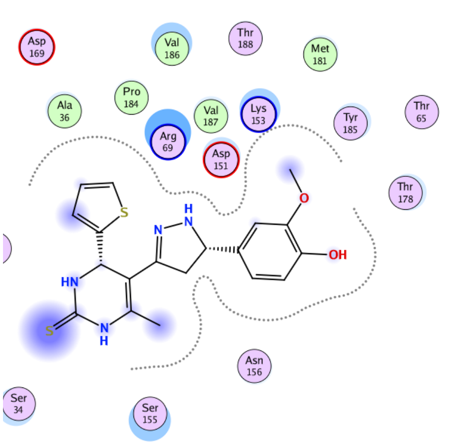

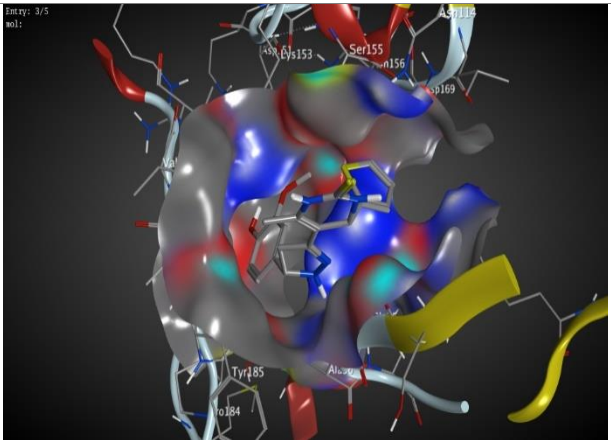

Figure S18: (Left side)5 2D and (right side) 3D representations of interactions of the docked novel synthesized pyrimidine-2-thione derivatives with amino acid residues of the H-RAS target protein using MOE docker software

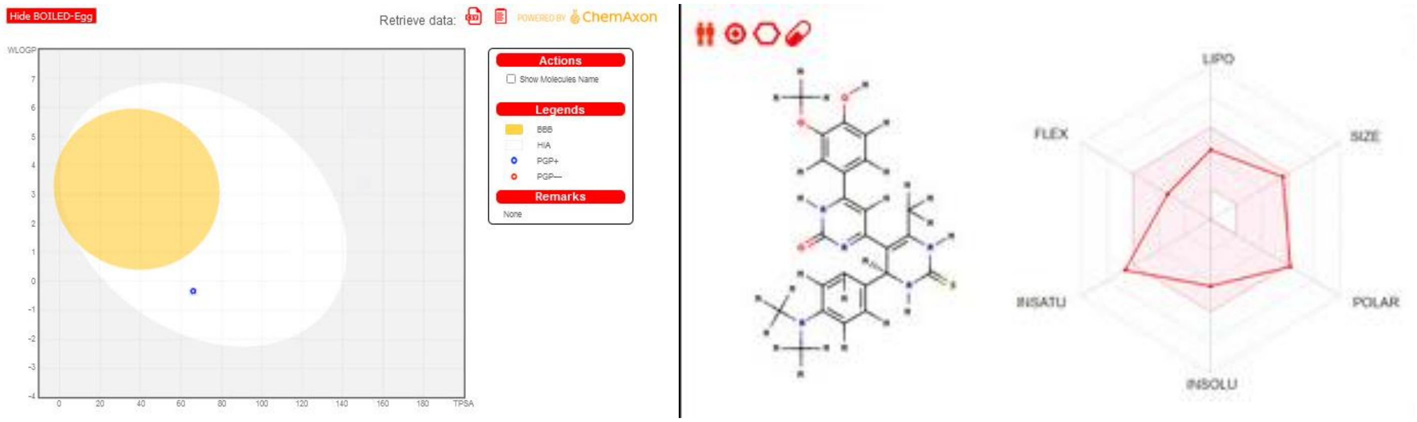

Figure S19: Bioavailability graphical data of 3a

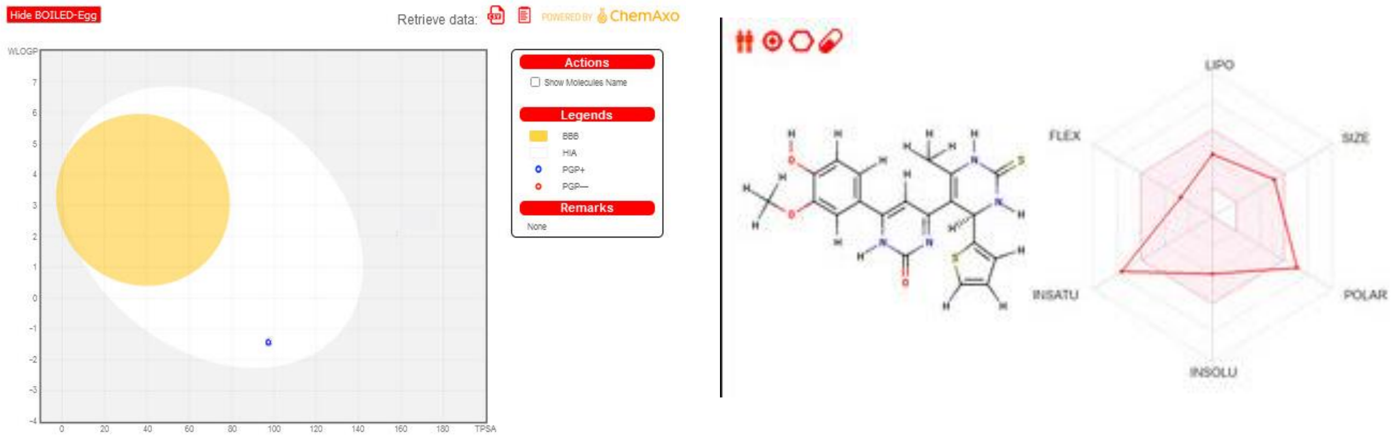

Figure S20:Bioavailability graphical data of 3b

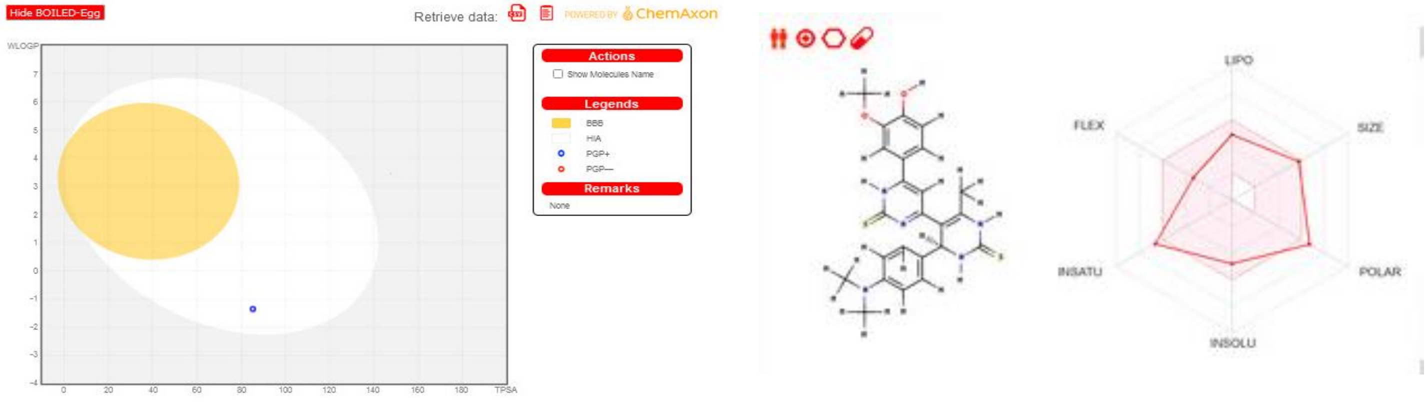

Figure S21: Bioavailability graphical data of 4a

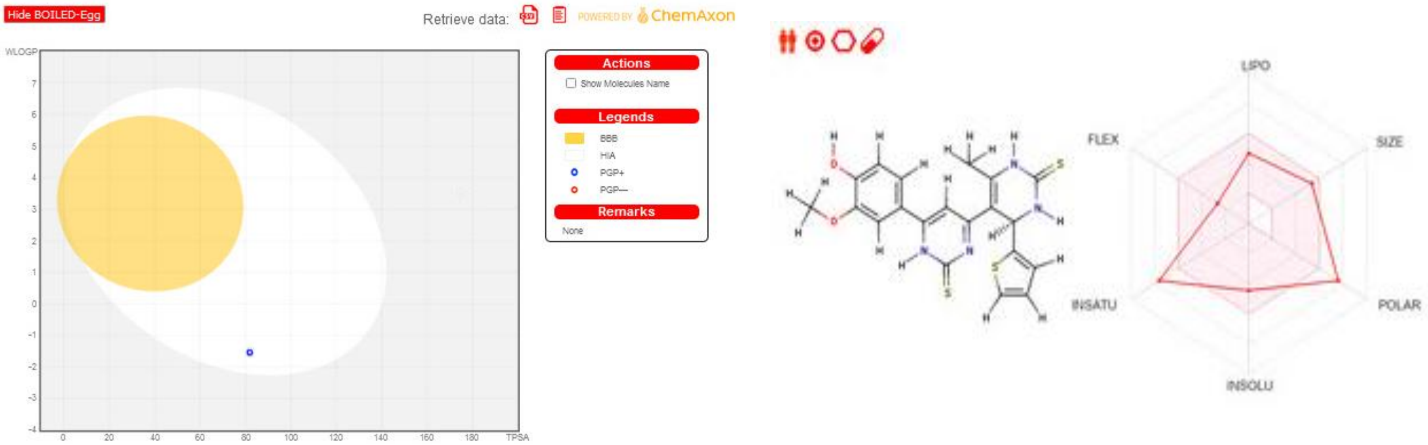

Figure S22:Bioavailability graphical data of 4b

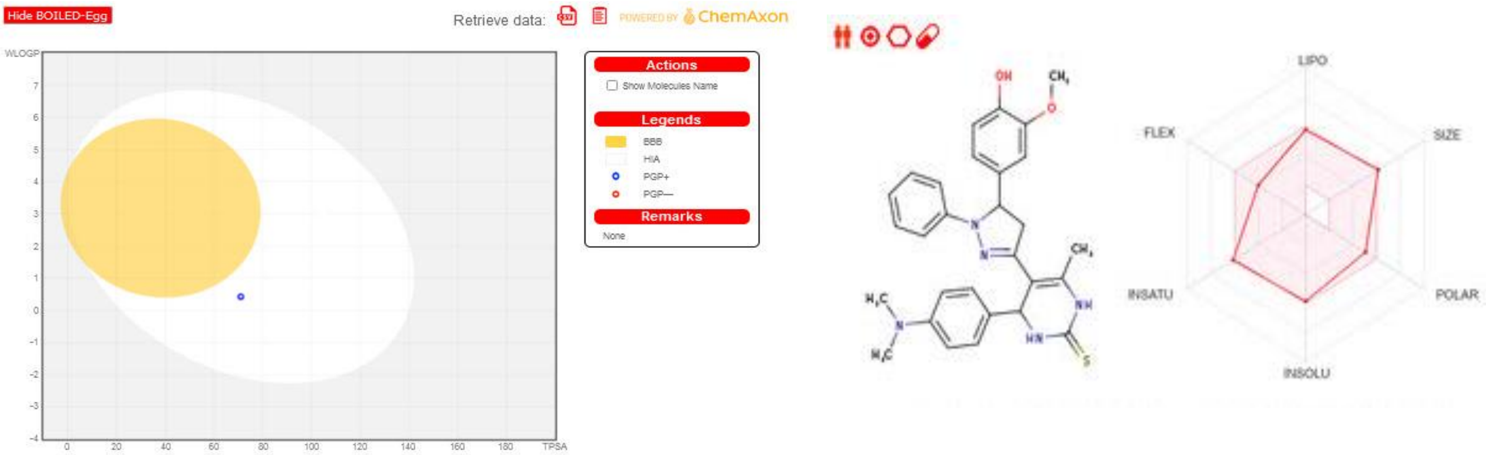

Figure S23:Bioavailability graphical data of 5a

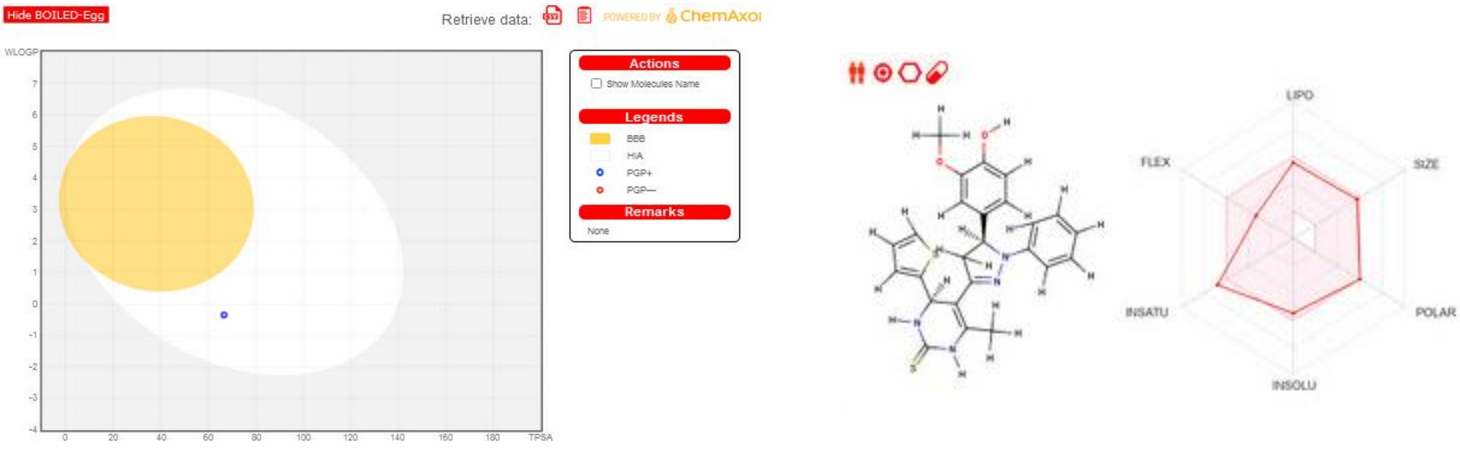

Figure S24: Bioavailability graphical data of 5b

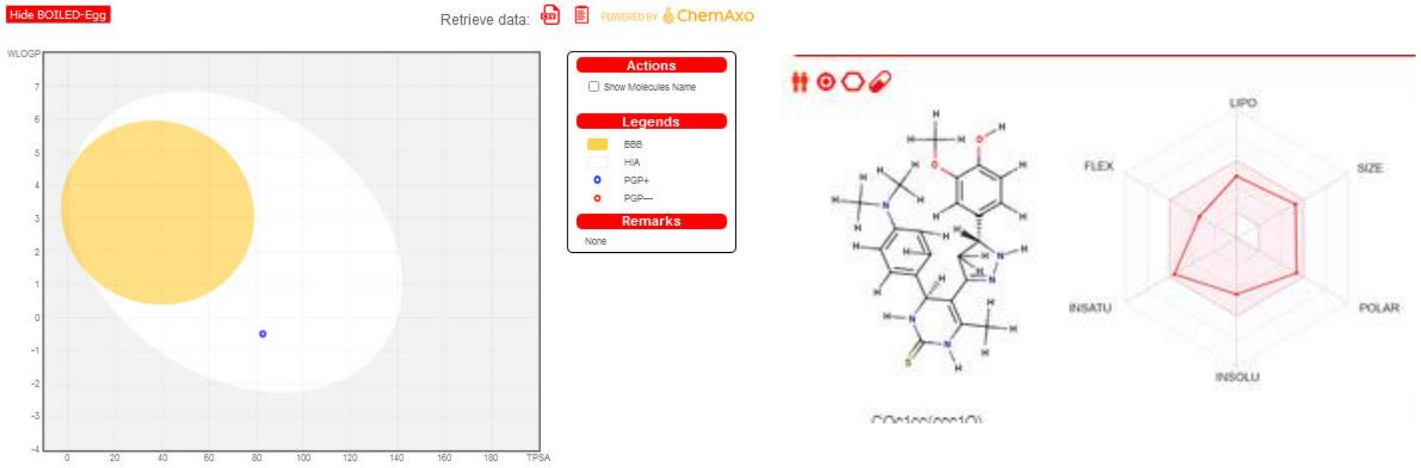

Figure S25: Bioavailability graphical data of 6a

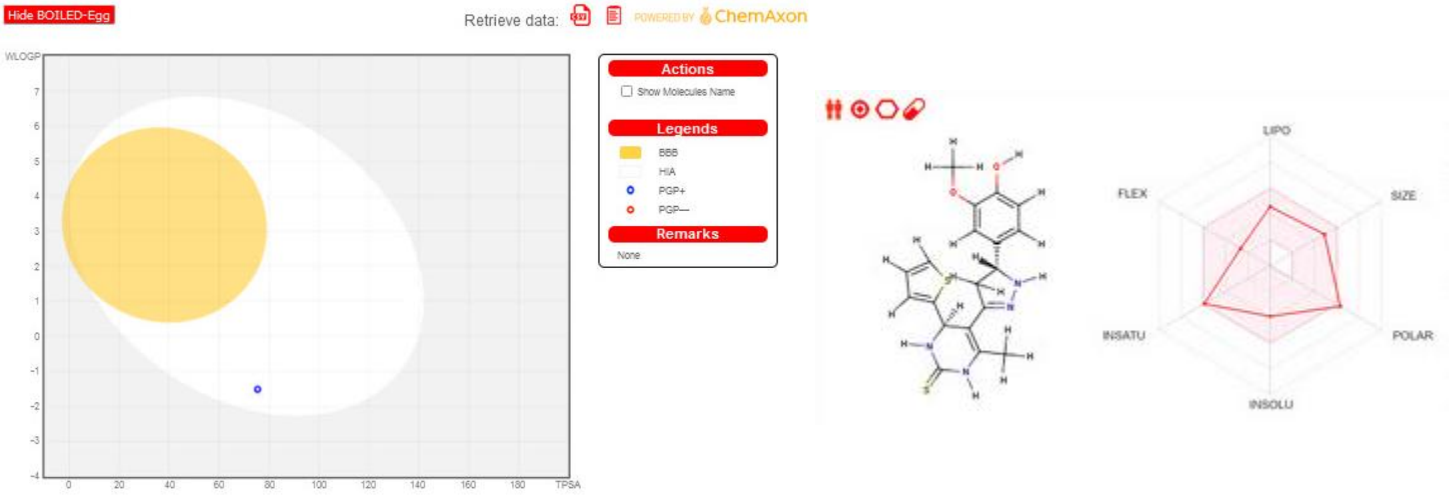

Figure S26: Bioavailability graphical data of 6b

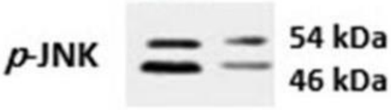

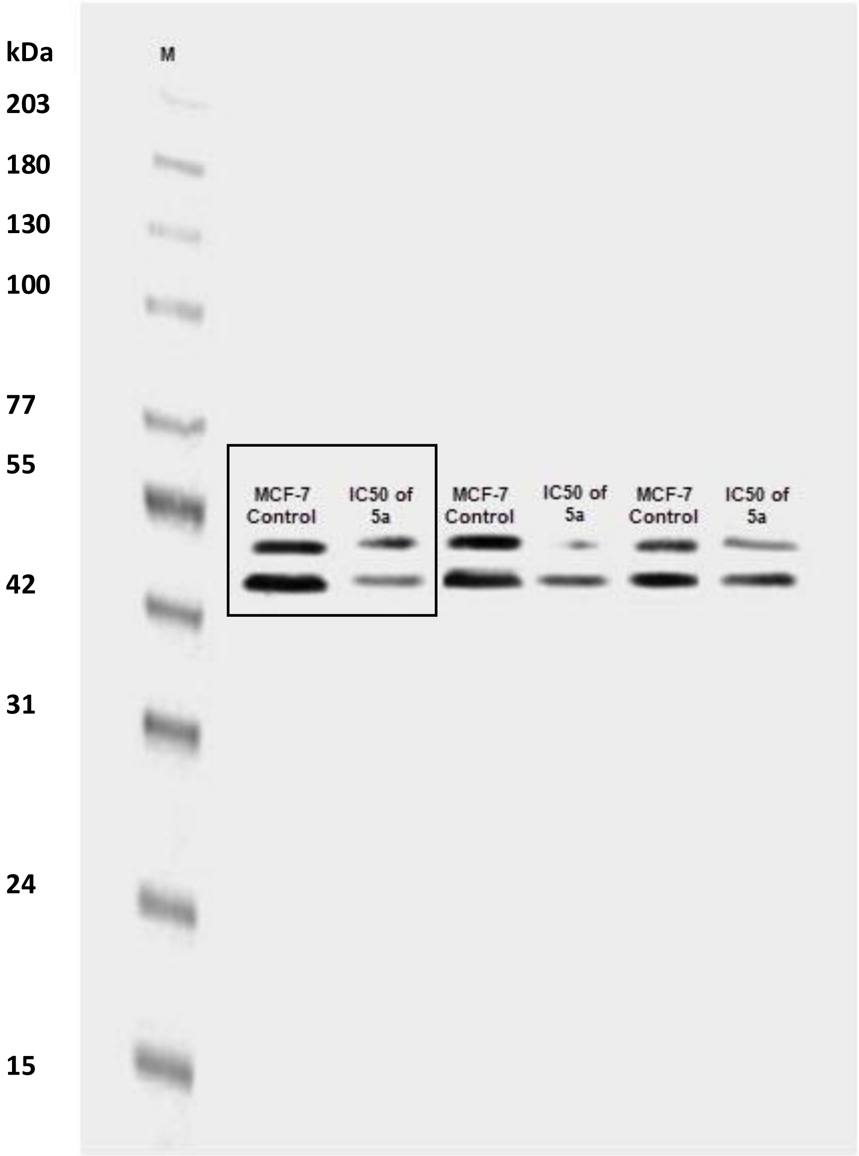

Figure S27: **Raw western blot gel of *p*-JNK (Whole mount western blot.)**

(In triplicate)

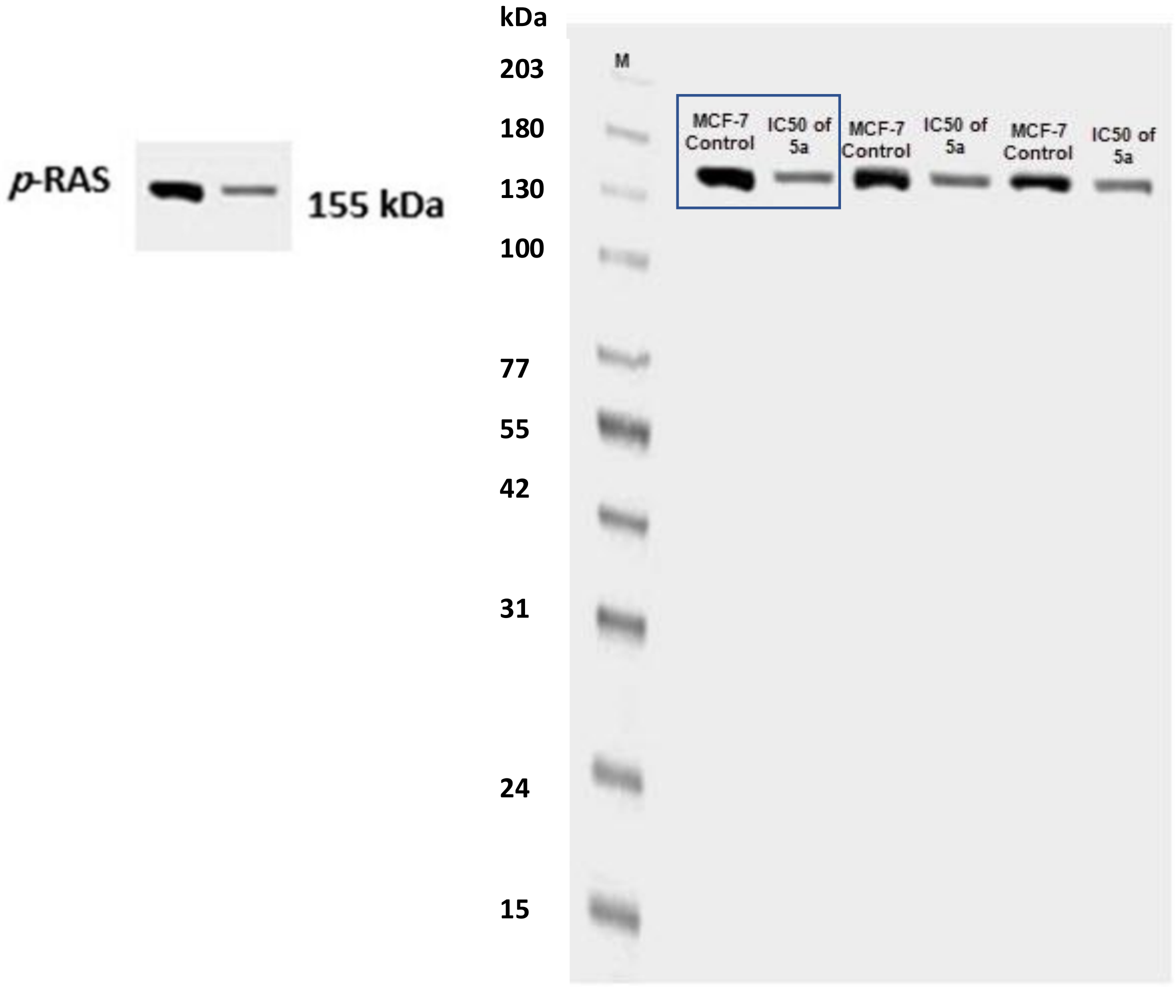

Figure S28: **Raw western blot gel of *p*-RAS (Whole mount western blot.)**

(In triplicate)

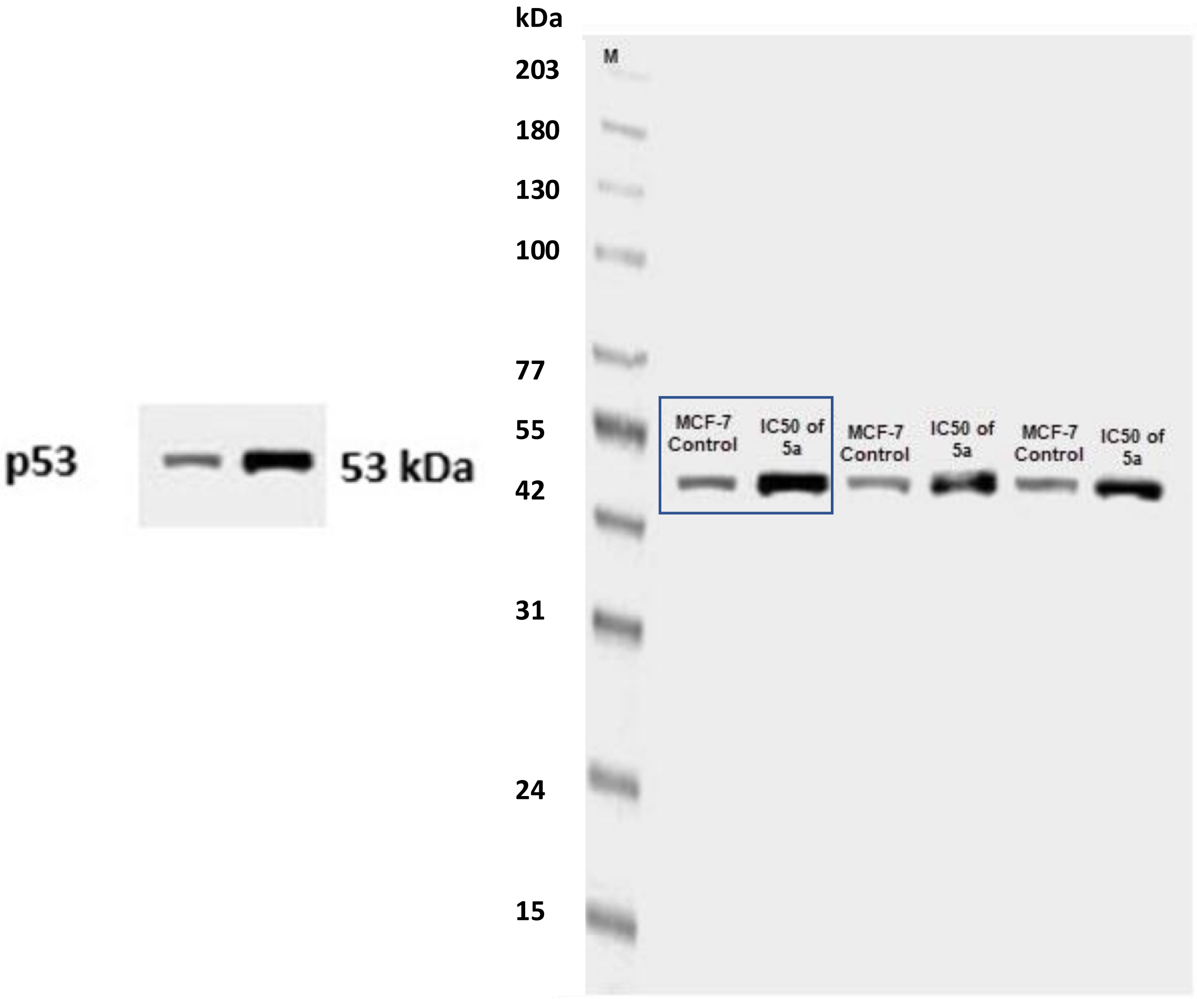

Figure S29: **Raw western blot gel of p53 (Whole mount western blot.)**

(In triplicate)

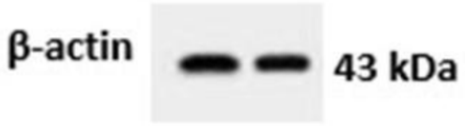

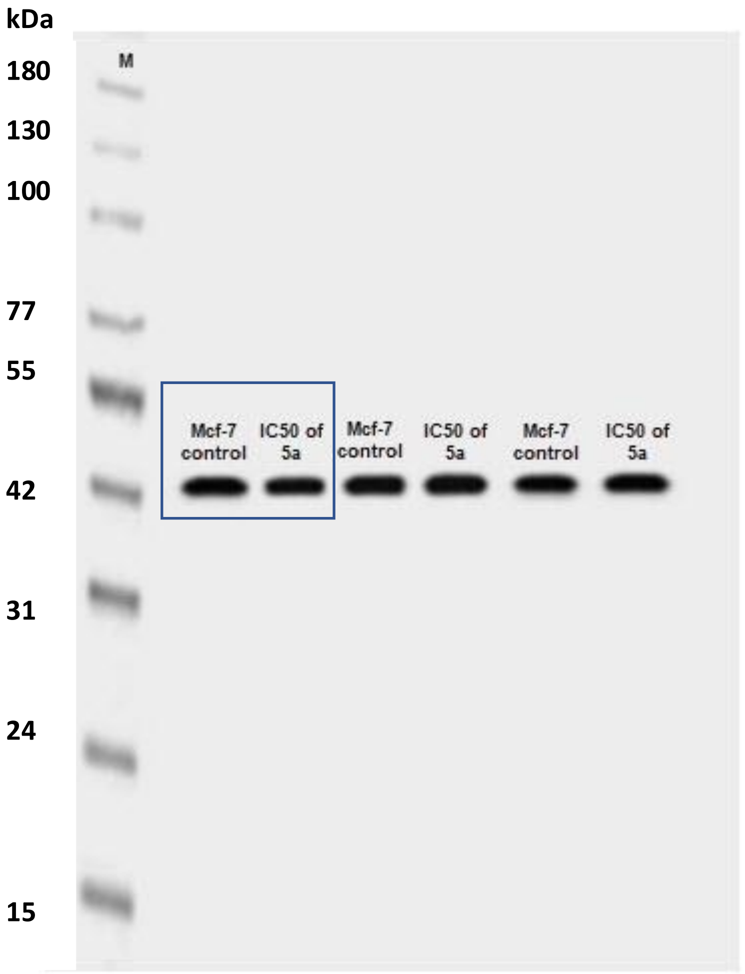

Figure S30: **Raw western blot gel of β-actin (Whole mount western blot.)**

(In triplicate)

General information

Melting points were measured using a Gallenkamp melting point apparatus (uncorrected). Fourier transform infrared (FT-IR) spectra were recorded on a Perkin-Elmer FTIR 1430 spectrophotometer using the KBr disk technique. In addition, ^{1}H nuclear magnetic resonance (NMR) spectra were recorded using a Bruker AC spectrometer (400 MHz) at 25°C in dimethyl sulfoxide (DMSO-*d6)* with tetramethylsilane (TMS) as an internal standard, and chemical shifts are reported in parts per million as d values; ^{13}C NMR was set at 101 MHz. The elemental analyses for C, H, N, and S were performed at the Regional Center for Mycology and Biotechnology, Al-Azhar University. Reaction progress was monitored via thin layer chromatography (TLC). We used an ultrasonic cleaner set (WUC-D03H) with a frequency and power of 60 Hz and 290 W, respectively, as a green chemistry technique.
